# Towards a quantitative assessment of inorganic carbon cycling in photosynthetic microorganisms

**DOI:** 10.1002/elsc.201900061

**Published:** 2019-10-31

**Authors:** Stefan Müller, Tomáš Zavřel, Jan Červený

**Affiliations:** ^1^ Faculty of Mathematics University of Vienna Wien Austria; ^2^ Department of Adaptive Biotechnologies Global Change Research Institute of the Czech Academy of Sciences Brno Czech Republic

**Keywords:** carbonate chemistry, computational modeling, cyanobacteria, futile cycles, photosynthesis

## Abstract

Photosynthetic organisms developed various strategies to mitigate high light stress. For instance, aquatic organisms are able to spend excessive energy by exchanging dissolved CO_2_ (dCO_2_) and bicarbonate (${\mathrm{HCO}}_{3}^{-}$) with the environment. Simultaneous uptake and excretion of the two carbon species is referred to as inorganic carbon cycling. Often, inorganic carbon cycling is indicated by displacements of the extracellular dCO_2_ signal from the equilibrium value after changing the light conditions. In this work, we additionally use (i) the extracellular pH signal, which requires non‐ or weakly‐buffered medium, and (ii) a dynamic model of carbonate chemistry in the aquatic environment to detect and quantitatively describe inorganic carbon cycling. Based on simulations and experiments in precisely controlled photobioreactors, we show that the magnitude of the observed dCO_2_ displacement crucially depends on extracellular pH level and buffer concentration. Moreover, we find that the dCO_2_ displacement can also be caused by simultaneous uptake of both dCO_2_ and ${\mathrm{HCO}}_{3}^{-}$ (no inorganic carbon cycling). In a next step, the dynamic model of carbonate chemistry allows for a quantitative assessment of cellular dCO_2_, ${\mathrm{HCO}}_{3}^{-}$, and H^+^ exchange rates from the measured dCO_2_ and pH signals. Limitations of the method are discussed.

**Abbreviations**
CA: carbonic anhydrase
dCO_2_: dissolved carbon dioxide
DIC: dissolved inorganic carbon
dO_2_: dissolved oxygen
ICC: inorganic carbon cycling
MIMS: membrane inlet mass spectrometry

## 1 INTRODUCTION

Living organisms developed numerous adaptations to gain advantages in diverse environments. One branch of metabolic adjustments, widely spread throughout Bacteria, Plantae, Fungi, and Animalia kingdoms, is the evolution of so‐called futile cycles. These cycles are part of regulation of the cellular energy status 1, 2, reproduction processes 3, primary or secondary metabolism 4, 5, 6, 7, redox components 8, signal transduction 9, and nutrient transport and assimilation pathways 10, 11, 12, 13, 14. Despite their name, futile cycles have specifically addressed functions in cellular metabolism, and the term “futile” may be misleading. The common feature of these cycles, namely energy loss, is advantageous in many cases. In photosynthetic organisms, controlled energy dissipation is essential for cellular homeostasis. Without photoprotection mechanisms, sudden energy income can cause irreversible destruction of the photosynthetic apparatus. Photosynthetic organisms developed several strategies for the dissipation of excessive light energy such as adjustments of light‐harvesting antennas, thermal energy dissipation, photo‐protective adjustments of the electron transport chain, reactive oxygen species scavenging 15, 16, 17, 18, 19, 20, and also light‐dependent “futile” inorganic carbon cycling (ICC) between cells and their aquatic environment 2, 21.

ICC, mediated through components of the carbon concentrating mechanism, can be defined as simultaneous uptake of dissolved carbon dioxide (dCO_2_) and excretion of bicarbonate (${\mathrm{HCO}}_{3}^{-}$), or vice versa simultaneous ${\mathrm{HCO}}_{3}^{-}$ uptake and dCO_2_ efflux. In cyanobacteria, ${\mathrm{HCO}}_{3}^{-}$ can enter the cells via the transporters BCT1, SbtA and BicA, and dCO_2_ by NDH‐I_3_ and NDH‐I_4_ 22, 23, 24. In eukaryotic microalgae, dissolved inorganic carbon (DIC) transporters comprise HLA3, LCIA, possibly LCI1 and CCP1/2 (in *Chlamydomonas reinhardtii*), SLC4 and its homologues (in diatoms), and possibly also other transporters in other species 22. Once inside the cell, dCO_2_ is reduced to ${\mathrm{HCO}}_{3}^{-}$ by carbonic anhydrase (CA) or CA‐like enzymes, and ${\mathrm{HCO}}_{3}^{-}$ is transported for DIC assimilation by RuBisCo either to the carboxysome (in cyanobacteria), the pyrenoid (in some algae) or elsewhere in the chloroplast (in other algae). In algae, more pathways for DIC transport to chloroplast are known, e.g. the transport of intermediates of the C4‐like metabolism 25. During ICC, part of DIC also leaves the cell. The DIC efflux pathways are far less understood than DIC uptake pathways. CO_2_ is known to diffuse out of the cell, either through membranes or pores, however, ${\mathrm{HCO}}_{3}^{-}$ excretion still remains an open question.

Although ${\mathrm{HCO}}_{3}^{-}$excretion transporters in cyanobacteria or algae have not yet been identified, it is assumed that ${\mathrm{HCO}}_{3}^{-}$can be excreted through anion channels. ${\mathrm{HCO}}_{3}^{-}$ channels have been found in plants and have also been proposed for microalgae; for a recent review, see ref. [22]. Both constitutively transcribed transporters BTC1 and HLA3 require ATP (whereas the transporters SbtA and BicA, induced by low inorganic carbon, require only Na^+^ export, mediated by H^+^/Na^+^ antiporters such as NhaS3). Further, the conversion of dCO_2_ to ${\mathrm{HCO}}_{3}^{-}$, mediated by CA‐like activity of NDH‐I_3/4_ (where NDH‐I_4_ is also transcribed constitutively), requires reduction equivalents such as NADPH or ferredoxin 23. Hence, a substantial amount of both energy and reductant equivalents formed under (excessive) light can be dissipated by ICC. A recent study also suggests that the CA‐like activity of NDH‐I (regulated through EcaB) prevents over‐reduction of plastoquinone pool under high light 26.

**PRACTICAL APPLICATION**

Quantification of carbon fluxes in aquatic photoautotrophic communities is of significant importance for understanding and accurate assessment of cellular carbon partitioning and carbon economy. In this study, we present methods for a quantitative description of inorganic carbon cycling between photosynthetic microorganisms and their environment. The often overlooked phenomena of carbon cycling performed by some photosynthetic microorganisms can negatively influence the accuracy of the measurement of carbon‐related properties of cells, e.g. their sequestration capacity, in dynamic culturing environments and can lead to incorrect bioenergetic calculations. Developed model and the technique for fast and accurate measurement of dissolved carbon dioxide provide a tool for identification and quantification of fast carbon fluxes that can be eventually used for smarter, model‐based, design of biotechnological solutions.

**Figure 1:**
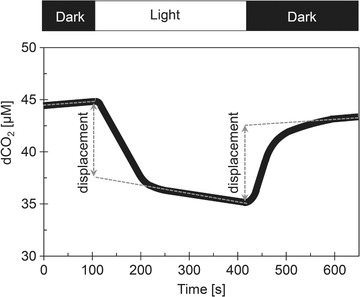
Representative example of a Dark‐Light‐Dark experiment to detect inorganic carbon cycling from the dCO_2_ signal, measured by a membrane inlet mass spectrometer. In the dark phases, dCO_2_ fluxes are dominated by cellular respiration which causes a net increase of dCO_2_, whereas in the light phase, dCO_2_ fluxes are dominated by carbon fixation which results in net dCO_2_ decrease. The abrupt dCO_2_ displacement at the beginning and at the end of the light phase is typically explained as the effect of ICC (in this case as simultaneous dCO_2_ uptake and ${\mathrm{HCO}}_{3}^{-}$ excretion), however, as discussed in the text, such a displacement can be observed even in the absence of ICC. The flat dCO_2_ slopes represent the net effects of dCO_2_ and ${\mathrm{HCO}}_{3}^{-}$ fluxes (between cells and the aquatic environment) close to equilibrium. Figure adapted from Tchernov et al., 2003 2

ICC can be detected from the dCO_2_ signal, measured by methods with high sensitivity and high temporal resolution, such as membrane inlet mass spectrometry (MIMS). A representative example of a Dark‐Light‐Dark experiment (to detect ICC) is shown in Figure 1; in the dark phases dCO_2_ increases mainly as a consequence of cellular respiration (although also carbon fixing processes can take place in the dark), whereas in the light phase dCO_2_ decreases mainly as a consequence of DIC assimilation (although CO_2_ producing processes such as respiration typically also take place at light). Most notably, the presence of ICC (in the case of Figure 1, simultaneous dCO_2_ uptake and ${\mathrm{HCO}}_{3}^{-}$ excretion) causes an abrupt displacement of dCO_2_ (a dynamics far from chemical equilibrium).

In this work, we show that in addition to the dCO_2_ signal, also the pH signal can be used for ICC detection. For quantitative description of DIC exchange rates between photosynthetic cells and the aquatic environment, we developed a dynamic carbonate chemistry model. The model allows for detection of ICC activity and quantification of DIC reaction rates, and it extends previously published model 27 by considering dynamics far from equilibrium. The results show that the presence of ICC is not trivial to detect, in particular, that the dCO_2_ displacement at the transitions between dark and light phases need not be caused by ICC. In fact, under high buffer concentrations such a displacement can be observed also in the absence of ICC. On the other hand, for low buffer concentrations and low pH (<7) it is not possible to detect ICC solely from the dCO_2_ signal, but only in combination with the pH signal. We validated our model predictions experimentally, using MIMS in combination with well‐controlled photobioreactors. For the assessment of ICC, we therefore recommend to measure dCO_2_ and pH simultaneously (with high accuracy and temporal resolution) and hence to use weak buffer concentrations in the cultivation media.

The model can be further used to quantitatively determine dCO_2_ and ${\mathrm{HCO}}_{3}^{-}$ exchange rates between photoautotrophic cells and the aquatic environment from experimental data. Our unique combination of a highly controlled experimental setup with a mathematical model can be used as a tool for detection and quantification of ICC in photoautotrophic microorganisms.

## 2 MATERIALS AND METHODS

### 2.1 A dynamic model of carbonate chemistry in the aquatic environment

For the purpose of (i) a quantitative description of dCO_2_, ${\mathrm{HCO}}_{3}^{-}$, and H^+^ exchange rates between photoautotrophic cells and the aquatic environment and (ii) a quantitative assessment of ICC in photosynthetic microorganisms, we developed a dynamic model of carbonate chemistry and related processes. The model comprises the following variables (and constants):

[CO_2_]: concentration of dCO_2_
[${\mathrm{HCO}}_{3}^{-}$]: concentration of bicarbonate ions
[H^+^]: concentration of protons
*CP*: carbonate pool; see Equation 3d
*TA*: total alkalinity; see Equation 3e
*q*CO_2_: CO_2_ exchange rate between cells and the aquatic environment
*q* ${\mathrm{HCO}}_{3}^{-}$: ${\mathrm{HCO}}_{3}^{-}$ exchange rate between cells and the aquatic environment
*q*H^+^: H^+^ exchange rate between cells and the aquatic environment
*v*: net hydration rate of dCO_2_; see Equation (2)
$c_{B}^{tot}$: concentration of buffer

**Figure 2:**
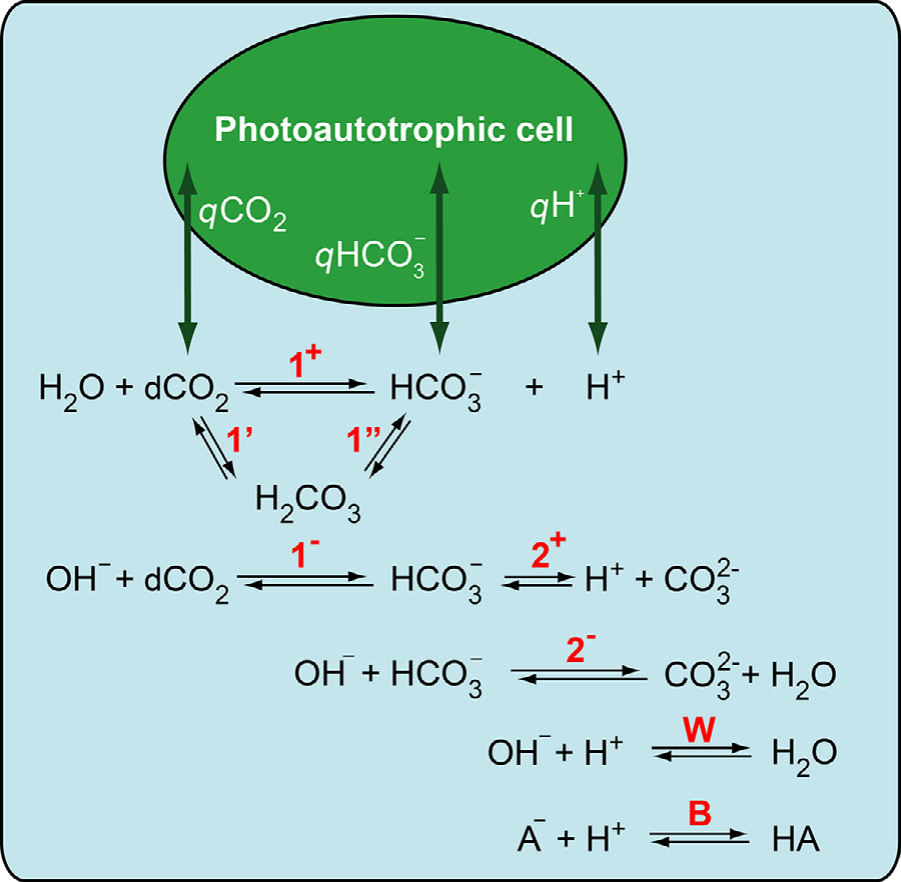
Reactions of carbonate species (together with water self‐dissociation and buffer) in the aquatic environment and dCO_2_, ${\mathrm{HCO}}_{3}^{-}$, and H^+^ exchange rates (called *q*CO_2_, *q* ${\mathrm{HCO}}_{3}^{-}$, and *q*H^+^) between photoautotrophic cells and aquatic environment. The overall hydration rate constant *k*_1_ is determined by the rate constants of the forward reactions 1′, 1^+^, and 1^−^; see Equation 1. Reactions 1″, 2^+^, 2^−^, W, and B are considered as fast and equilibrium is assumed; see Table 1 for the equilibrium constants *K*_1_, *K*_2_, *K*_W_, and *K*_B_. Figure adapted from Nedbal et al., 2010 27

The carbonate chemistry reactions are summarized in Figure 2, the corresponding rate and equilibrium constants are summarized in Table 1 and the resulting dynamic model is presented in Equations 3a–e. The model derivation and analysis is provided in the Supporting Information.

**Table 1:** Rate and equilibrium constants, as measured in the cultivation medium BG‐11, together with reference constants for sea water

		Value			Reference	
Constant	Comment	BG‐11	Sea water	Unit	BG‐11	Sea water
*k*_1_	dCO_2_ hydration rate constant	0.04	0.04	[s^−1^]	Measured	42
*K*_1_	Equilibrium constant of reaction dCO_2_ + H_2_O ⇌ ${\mathrm{HCO}}_{3}^{-}$ + H^+^	10^−6.30^	10^−5.86^	[mol L^−1^]	Measured	42
*K*_2_	Equilibrium constant of reaction ${\mathrm{HCO}}_{3}^{-}$ ⇌ ${\mathrm{CO}}_{3}^{2-}$ + H^+^	10^−9.00^	10^−8.92^	[mol L^−1^]	Measured	42
*K*_W_	Water self‐dissociation constant	10^−14.00^	10^−13.22^	[mol L^−1^]	42	42
*K*_B_	Equilibrium constant of HEPES buffer	10^−7.55^		[mol L^−1^]		

Reactions 1″, 2^+^, 2^−^, W, and B of carbonate species, as shown in Figure 2, are considered as fast, and chemical equilibrium is assumed (as determined by the equilibrium constants *K*_1_, *K*_2_, *K*_W_, and *K*_B_ as summarized in Table 1). Reactions 1′, 1^+^, and 1^−^ are considered slow, and the resulting overall hydration rate constant *k*_1_ is given by:

(1) $$k_{1}=k_{1^{\prime }}^{⇀}+k_{1^{+}}^{⇀}+k_{1^{-}}^{⇀}\frac{K_{W}}{\left[H^{+}\right]}$$

As a consequence, the net hydration rate of dCO_2_ (determined by the slow reactions 1′, 1^+^, and 1^−^) can be written as:

(2) $$v=k_{1}\left(\left[CO_{2}\right]-\left[{\mathrm{HCO}}_{3}^{-}\right]\frac{\left[H^{+}\right]}{K_{1}}\right)$$

After introducing the carbonate pool *CP* and total alkalinity *TA*, we can present our dynamic model for the five variables [CO_2_], [${\mathrm{HCO}}_{3}^{-}$], [H^+^], CP, TA (for given exchange rates *q*CO_2_, *q* ${\mathrm{HCO}}_{3}^{-}$, and *q*H^+^). The differential algebraic equation system contains three ordinary differential equations and two algebraic equations:

(3a) $$\frac{d[CO_{2}]}{dt}=-v+qCO_{2}$$

(3b) $$\frac{\mathrm{dCP}}{dt}=+v+{q\mathrm{HCO}}_{3}^{-}$$

(3c) $$\frac{dTA}{dt}=+{q\mathrm{HCO}}_{3}^{-}-qH^{+}$$

(3d) $$CP=\left[{\mathrm{HCO}}_{3}^{-}\right]\left(1+\frac{K_{2}}{\left[H^{+}\right]}\right)$$

(3e) $$\begin{array}{ccc} TA & = & \left[{\mathrm{HCO}}_{3}^{-}\right]\left(1+\frac{2K_{2}}{\left[H^{+}\right]}\right)-\left[H^{+}\right]\\ & & +\frac{K_{W}}{H^{+}}+c_{B}^{tot}\frac{1}{1+\frac{\left[H^{+}\right]}{K_{B}}} \end{array}$$

Further details including model derivation as well as model analysis are provided in the Supporting Information. For numerical solutions of the differential algebraic equation system (3), we used *Wolfram Mathematica 10* (Wolfram Research, Champaign, IL, USA) in particular, the function *NDSolve* with default options.

### 2.2 Experimental setup (for validating the model predictions)

#### 2.2.1 Inoculum culture conditions

Model predictions were validated experimentally, using a cyanobacterium *Synechocystis* sp. PCC 6803. The strain was kindly provided by Dr. Martin Hagemann. The inoculum cultures were pre‐cultivated in 250 mL Erlenmeyer flasks on air on a standard orbital shaker at 31°C, under 110 µmol(photons) m^−2^ s^−1^ of warm white LED light and in cultivation medium BG‐11 28 supplemented with 17 mM HEPES (Sigma‐Aldrich, St. Louis, MO, USA). Prior to the experiments, cells from Erlenmeyer flask (c. 100 mL) were centrifuged (2000 *x g*, 10 min), supernatant was discarded, and pellet was washed two times with HEPES‐free BG‐11 medium, to ensure minimal buffer concentration in the culture media. The culture was then inoculated into the photobioreactor.

#### 2.2.2 Photobioreactor

For the validating experiments, we used a flat‐panel photobioreactor described in detail in 29. The bioreactor illumination was secured by red and blue LED panels with chess board LEDs configuration (red: λ_max_ ≈ 633 nm, Δλ_1/2_ ≈ 20 nm, Luxeon LXHL‐PD09; blue: λ_max_ ≈ 445 nm, Δλ1/2 ≈ 20 enm, Luxeon LXHL‐PR09; both manufactured by Future Lighting Solutions, Montreal, QC, CA). The culture pH was continuously monitored by InPro3253 electrode (Mettler‐Toledo, 1900 Polaris Parkway, Columbus, OH 43240, USA), the own‐developed scripts allowed measurement of pH in temporal resolution of 1 s. Culture temperature was monitored by the same electrode and controlled by a Peltier cell incorporated in the instrument base. All other photobioreactor accessories and properties were such as described in Sinetova et al., 2012 30.

#### 2.2.3 Gas exchange rate measurement

Dynamics in dCO_2_ and dissolved oxygen (dO_2_) exchange between cells and cultivation media was measured by MIMS, described in detail in Zavřel et al., 2016 31. Briefly, MIMS consisted of three main parts: (i) the mass spectrometer (PrismaPlus™ QMG 220 M1 with open ion source, Pfeiffer Vacuum, Asslar, Germany), (ii) silicon membrane at the system inlet (SILASTIC 508‐006, Dow Corning, Midland, MI, USA), and (iii) tubing between the membrane inlet and the analyzer; stainless steel tubing connected with 90° or 180° stainless steel fittings (all manufactured by Swagelok, Solon, Ohio, USA) with U‐shape water trap constantly tempered to −100 to −80°C for capturing water molecules crossing the silicone membrane, according to Tu et al., 1987 32. The MIMS was configured to detect m/z 32 (O_2_), 44 (CO_2_), and 40 (Ar).

#### 2.2.4 Carbonate system parametrization experiments

Equilibrium constants *K*_1_ and *K*_2_ of BG‐11 cultivation medium at 30°C were determined using high‐sensitivity Total Organic Carbon Analyzer (TOC‐V_CSH_, Shimadzu Scientific Instruments, Kyoto, Japan). All experiments for carbonate system parametrization were performed in a highly controlled environment of the photobioreactor to secure accurate and stable conditions, and the carbon analyzer was calibrated using certified premium range standards (TOC/TIC Standards, Reagecon Diagnostics, Shannon, Ireland).

First, to determine concentration of the dCO_2_ in BG‐11 medium, we adjusted pH of BG‐11 medium to pH 4.0 by addition of 1 M hydrochloric acid and aerated the medium with 15 000 ppm CO_2_ + air mixture (calibration cylinder 15 000 ± 75 ppm of CO_2_ in dry air, SIAD Czech, Rajhradice, Czechia) long enough to eliminate ${\mathrm{HCO}}_{3}^{-}$; the bicarbonate elimination was validated by sequential sampling for total inorganic carbon quantification in the time interval of several hours.

The equilibrium constant *K*_1_ (relating dCO_2_ and ${\mathrm{HCO}}_{3}^{-}$) was determined from a series of measurements in BG‐11 medium for slightly acidic to neutral pH levels, adjusted by addition of 1 M hydrochloric acid, following similar approach as for determination of dCO_2_; at each pH level the medium was aerated with 15 000 ppm CO_2_ + air mixture and the total inorganic carbon content was measured when the carbonate system equilibrated.

Finally, the equilibrium constant *K*_2_ (relating ${\mathrm{HCO}}_{3}^{-}$ and ${\mathrm{CO}}_{3}^{2-}$) was determined from a series of measurements in BG‐11 medium under slightly alkaline pH levels, adjusted by addition of 1 M sodium hydroxide. Calculated equilibrium constants according to the carbonate system description (Figure 2 and Supporting Information Section 1.2) are summarized in Table 1.

#### 2.2.5 Validating experiments

The experiments validating the model predictions were performed in a photobioreactor cuvette. Cells in non‐buffered BG‐11 cultivation media were inoculated into the cuvette, and kept at pH 8.5 and modest irradiance of 10 µmol(photons) m^−2^ s^−1^ of both red and blue light for 24 h for acclimation. The initial cell density was approximately 3 × 10^7^ cells mL^−1^. Temperature was stabilized at 30°C during all experiments.

The dynamics in dO_2_, dCO_2_, and pH during Dark‐Light‐Dark experiments was measured under pH levels 6.5, 7.3, 7.7, and 8.1. After 24 h of acclimation period, pH in the photobioreactor was decreased to 6.5 by addition of 1 M hydrochloric acid. dCO_2_ formed from ${\mathrm{HCO}}_{3}^{-}$ pool was bubbled out by air until the dCO_2_ concentration in the culture reached approximately 30 µM. Then, simultaneous measurement of gas exchange and pH was initiated. Each measurement consisted of three steps: (i) initial measurement in dark, (ii) measurement of photosynthetic activity at light, and (iii) final measurement in dark. Each step took 4 min.

Between the measurements, culture homogenization was secured by air bubbling through the photobioreactor cuvette (flow rate 200 mL min^−1^). During the measurements, bubbling was turned off and culture homogenization was secured by rotation of magnetic stirrer bar (ø5 × 35 mm) in a vertical plane (500 rpm). To secure light saturated conditions for the cells 33, light intensity during each measurement was set to 150 µmol(photons) m^−2^ s^−1^ of red light and 900 µmol(photons) m^−2^.s^−1^ of blue light. Between the measurements, the intensity of both lights was reduced to 10 µmol(photons) m^−2^ s^−1^. Once each measurement was completed, pH of the culture was increased by addition of 1 M sodium hydroxide to the next pH level (6.5–7.3–7.7–8.1). After pH increase, culture was bubbled with air until dCO_2_ level reached approximately 30 µM. Stabilization of pH took no longer than 45 min after each induced pH increase.

## 3 RESULTS

### 3.1 Model predictions

#### 3.1.1 Magnitude of the dCO_2_ displacement during inorganic carbon cycling strongly depends on buffer concentration and pH level

Using the dynamic model of carbonate chemistry, we can simulate dCO_2_ and pH signals dynamics under diverse conditions. By specifying the exchange rates between cells and their aquatic environment (*q*CO_2_, *q* ${\mathrm{HCO}}_{3}^{-}$, and *q*H^+^), both dark respiration and light‐dependent carbon fixation (as a sum of all dCO_2_, ${\mathrm{HCO}}_{3}^{-}$, and H^+^ fluxes between cells and their aquatic environment) can be simulated together to abrupt dCO_2_ displacements at the beginning and at the end of the light phase as caused by inorganic carbon cycling (Figure 1).

First, we studied the visibility of both dCO_2_ and pH displacements under standard laboratory conditions. In previous publications related to ICC, strongly buffered cultivation media with slightly alkaline pH was used 2, 34. In our simulations, we considered two scenarios: a highly buffered system ($c_{B}^{tot}$ = 17 mM 21, 33, 35), but also a weakly buffered system ($c_{B}^{tot}$ = 1 µM). In dark, we assumed excretion of both dCO_2_ and ${\mathrm{HCO}}_{3}^{-}$ from the cells to the cultivation media due to dark respiration. Under light, we assumed carbon fixation together with ICC, where dCO_2_ is assimilated and ${\mathrm{HCO}}_{3}^{-}$ is excreted from the cells to the cultivation medium. To preserve charge balance, we assumed *q*H^+^ = *q* ${\mathrm{HCO}}_{3}^{-}$, which results in constant total alkalinity *TA*, see Equation 3c.

**Figure 3:**
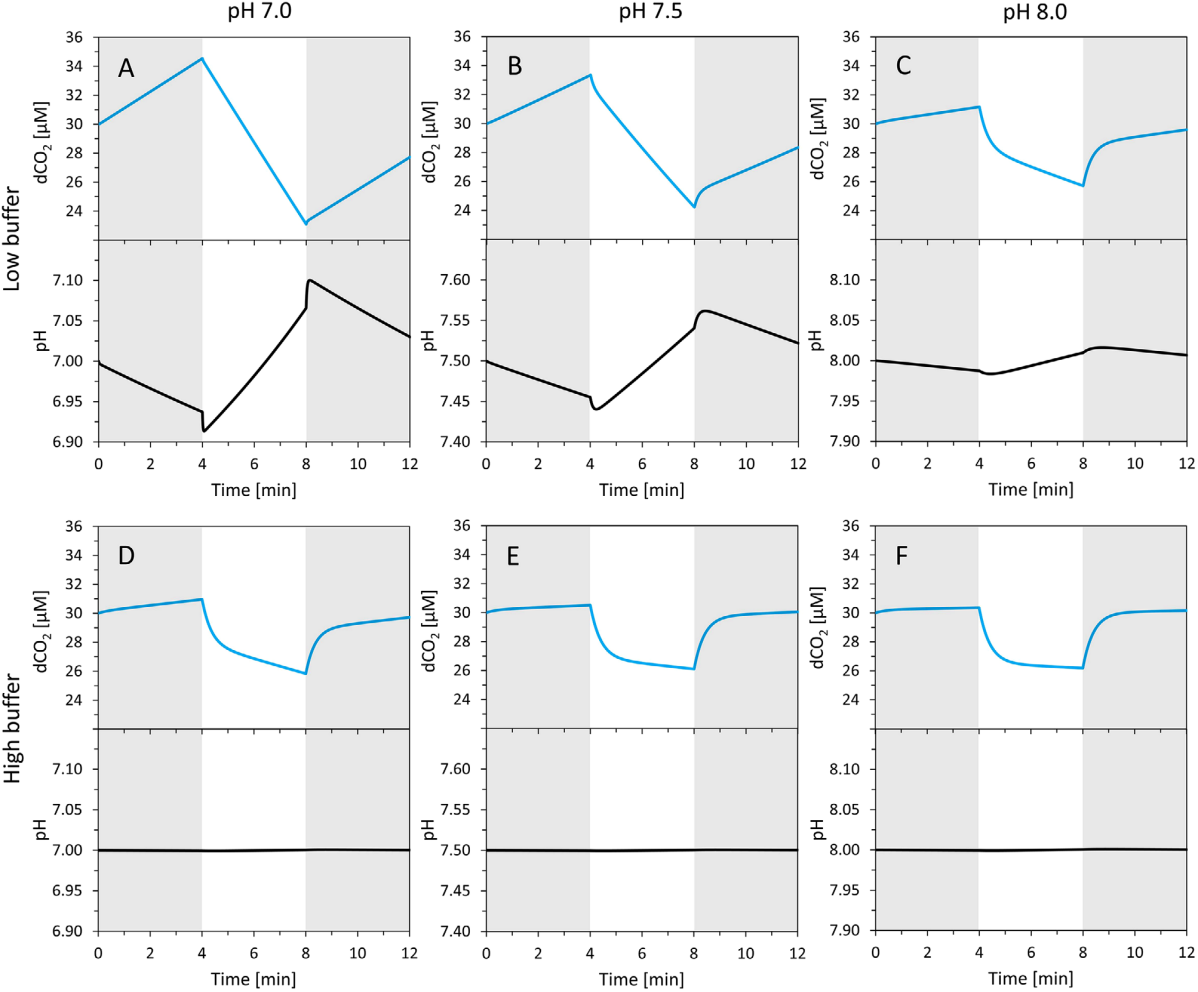
Simulations of dCO_2_ (blue lines) and pH (black lines) signals dynamics in the presence of inorganic carbon cycling, for initial pH 7.0 (left panels A, D), 7.5 (middle panels B, E), and 8.0 (right panels C, F), with buffer (HEPES) concentration of 1 µM (upper panels A, B, C) and 17 mM (lower panels D, E, F) during Dark‐Light‐Dark experiments. Dark phases (gray rectangles): exchange rates *q*CO_2_ and *q* ${\mathrm{HCO}}_{3}^{-}$ both set to +10 nmol L^−1^ s^−1^ (DIC excretion). Light phases: exchange rates set to −150 nmol L^−1^ s^−1^ for *q*CO_2_ (uptake) and +100 nmol L^−1^ s^−1^ for *q* ${\mathrm{HCO}}_{3}^{-}$ (excretion). Initial dCO_2_ concentration set to 30 µM

More specifically, we simulated dynamics in dCO_2_ and pH signals for pH levels of 7, 7.5, and 8 and under low and high buffer concentrations. Under light, the exchange rate *q*CO_2_ was set to −150 nmol L^−1^ s^−1^ (CO_2_ uptake), whereas *q* ${\mathrm{HCO}}_{3}^{-}$ was set to +100 nmol L^−1^ s^−1^ (${\mathrm{HCO}}_{3}^{-}$ excretion). The assumed magnitudes of the exchange rates *q*CO_2_ and *q* ${\mathrm{HCO}}_{3}^{-}$ are based on the previously published experimental data 36, 37, 38. Results of the simulations show that with low buffer concentration ($c_{B}^{tot}$ = 1 µM), the magnitude of the dCO_2_ displacement decreases with decreasing pH (from 8 to 7), although identical exchange rates *q*CO_2_ and *q* ${\mathrm{HCO}}_{3}^{-}$ are assumed (Figure 3A–C). Under low buffer, the simulations also predict an increasing pH displacement with decreasing pH level, as a consequence of decreasing ${\mathrm{HCO}}_{3}^{-}$ concentration in the aquatic environment under decreasing pH (Figure 3A–C). On the other hand, under high buffer ($c_{B}^{tot}$ = 17 mM), the magnitude of the dCO_2_ displacement remains almost constant (in the pH range 7–8), and the pH signal is shifted only negligibly (Figure 3D–F).

Model predictions of dCO_2_ signal dynamics under exchange rates *q*CO_2_ and *q* ${\mathrm{HCO}}_{3}^{-}$ ranging between −150 to +100 nmol L^−1^ s^−1^ (simulating uptake or excretion of both dCO_2_ and ${\mathrm{HCO}}_{3}^{-}$) are summarized in Supporting Information Figure S1.

#### 3.1.2 dCO_2_ displacement need not be caused by inorganic carbon cycling in strongly buffered systems

**Figure 4:**
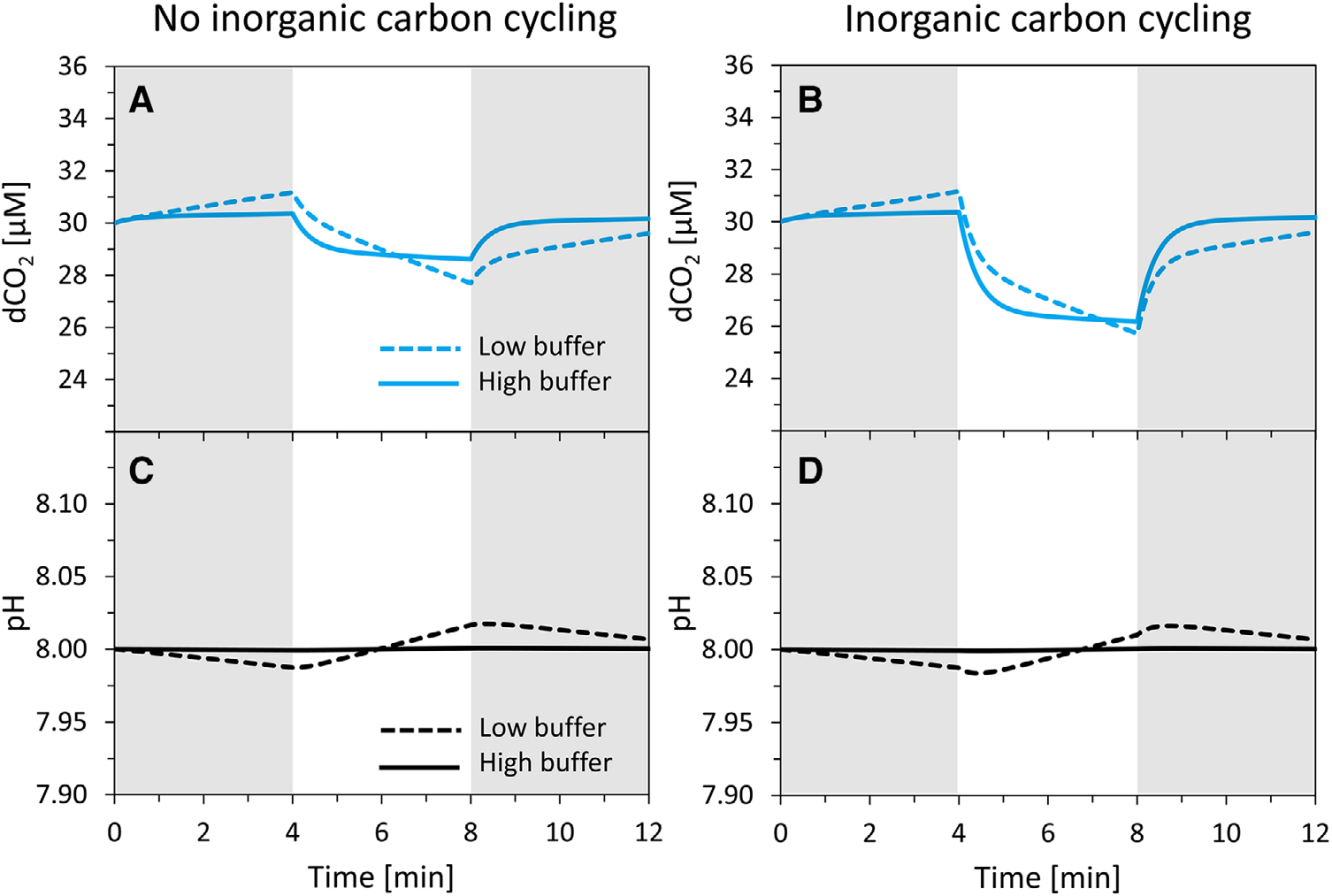
Simulations of dCO_2_ (blue lines) and pH (black lines) signal dynamics during Dark‐Light‐Dark experiments in absence (left panels A, C) and presence (right panels B, D) of inorganic carbon cycling (ICC) at initial pH 8.0, with HEPES concentration of 1 µM (dashed lines) and 17 mM (full lines). Dark phases (gray rectangles): exchange rates *q*CO_2_ and *q* ${\mathrm{HCO}}_{3}^{-}$ both set to +10 nmol L^−1^ s^−1^. Light phases (white rectangles): in absence of ICC, exchange rates set to −50 nmol L^−1^ s^−1^ for *q*CO_2_ (uptake) and 0 nmol L^−1^ s^−1^ for *q* ${\mathrm{HCO}}_{3}^{-}$; in presence of ICC, exchange rates set to −150 nmol L^−1^ s^−1^ for *q*CO_2_ (uptake) and +100 nmol L^−1^ s^−1^ for *q* ${\mathrm{HCO}}_{3}^{-}$ (excretion). Initial dCO_2_ concentration set to 30 µM

Figure 3 demonstrates the effect of pH and buffer on the visibility of dCO_2_ displacement in the presence of ICC. Figure 4 shows that with high buffer and initial pH 8.0 a visible dCO_2_ displacement can be observed even in the absence of ICC — in the case of sole carbon fixation where both dCO_2_ and ${\mathrm{HCO}}_{3}^{-}$ are taken up by the cells. This effect can be explained by analyzing the dynamics of dCO_2_ hydration which brings the system back (close) to equilibrium. As the model analysis shows (see Supporting Information Section 1.4), the net dCO_2_ hydration rate *v* (Equation 2) does not directly follow the hydration rate constant *k*_1_ (Equation 1), but the “apparent” dCO_2_ hydration rate defined as *k*_1_(1+α); see Supporting Information Equation 4. The parameter α ≥ 0 depends on buffer concentration and pH in a complicated way (Supporting Information Section 1.4 and Figure S2). The highest value of α (with α ≫ 1) and thus the fastest hydration dynamics is achieved at zero buffer concentration and pH 6.3 (equal to p*K*_1_). The lowest value α ≈ 0 and thus the slowest dynamics occurs for high buffer concentration and alkaline pH. Only then, the net hydration rate *v* follows the hydration rate constant *k*_1_, and the slow dynamics of the system returning back (close) to equilibrium is visible as a dCO_2_ displacement. The dynamics of the dCO_2_ hydration rate *v* during Dark‐Light‐Dark experiments (caused by shifts of dCO_2_, ${\mathrm{HCO}}_{3}^{-}$, and H^+^ exchange rates; see Supporting Information Equations 2 and 5 for further details) under pH 7.0 and 8.0 as well as under low and high buffer is summarized in Supporting Information Figure S3.

**Figure 5:**
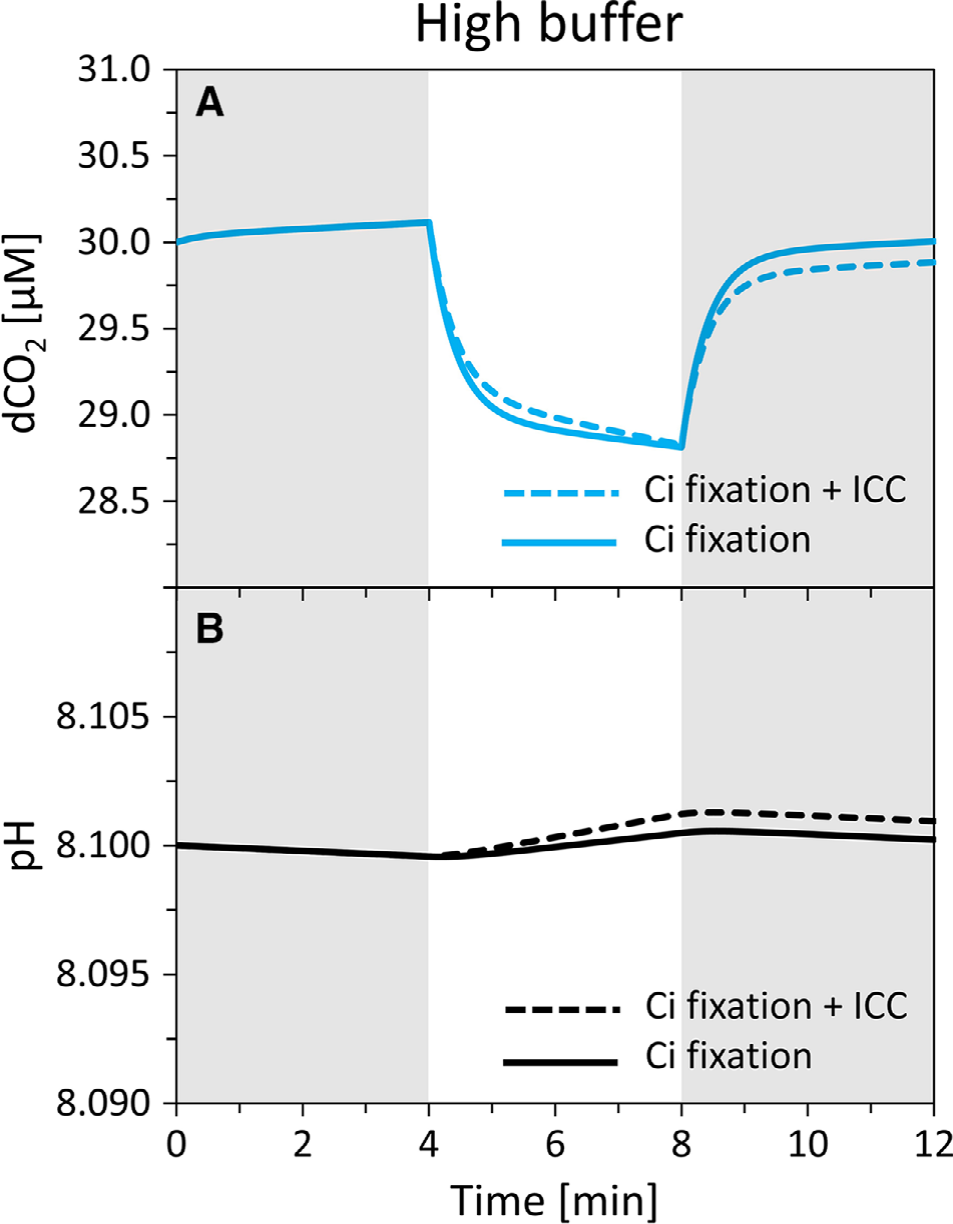
Simulations of similar dCO_2_ (blue lines) and pH (black lines) signals dynamics during Dark‐Light‐Dark experiments in absence of inorganic carbon cycling (ICC, dashed lines) and presence of ICC (full lines), with initial pH set to 8.1, initial dCO_2_ concentration set to 30 µM, and with buffer (HEPES) concentration set to 17 mM. Dark phases (gray rectangles): exchange rates set to +2 nmol L^−1^ s^−1^ for *q*CO_2_ and +10 nmol L^−1^ s^−1^ for *q* ${\mathrm{HCO}}_{3}^{-}$. Light phases: in absence of ICC (fixation only), exchange rates set to −40 nmol L^−1^ s^−1^ for *q*CO_2_ and −10 nmol L^−1^ s^−1^ for *q* ${\mathrm{HCO}}_{3}^{-}$ (uptake); in presence of ICC, exchange rates set to −45 nmol L^−1^ s^−1^ for *q*CO_2_ (uptake) and +15 nmol L^−1^ s^−1^ for *q* ${\mathrm{HCO}}_{3}^{-}$ (excretion)

As a consequence, under high buffer, it can be hard or even impossible to distinguish between sole carbon fixation and carbon fixation with ICC, when only measuring dCO_2_. In Figure 4, we set *q*CO_2_ and *q* ${\mathrm{HCO}}_{3}^{-}$ such that the resulting DIC fixation rate was −(*q*CO_2_ + *q* ${\mathrm{HCO}}_{3}^{-}$) = −50 nmol L^−1^ s^−1^, both in absence and presence of ICC. In Figure 5, we show that by choosing different DIC fixation rates in absence and presence of ICC, almost identical dCO_2_ dynamics can be obtained. Indeed, the particular choice of exchange rates *q*CO_2_ and *q*HCO_3_^−^ as used in Figure 5 is only one possible combination out of many.

The model can also be used to simulate the effect of various buffers (with different pKa values) on the visibility of the dCO_2_ displacement. In the simulations summarized in Supporting Information Figure S4, we considered buffers with pKa ranging from 5.59 (piperazine) to 10.51 (CAPS). In the presence of ICC, the dCO_2_ displacement is almost identical for all buffers, whereas in the absence of ICC, the dCO_2_ displacement decreases with an increasing difference between the pKa value of a particular buffer from the initial pH value of 8 considered in the simulation.

Based on the results of the simulations, we conclude that optimal conditions for the detection of ICC as well as for identifying the exchange rates *q*CO_2_ and *q* ${\mathrm{HCO}}_{3}^{-}$ from experimental data (see Section 3.5), weak pH buffer and simultaneous monitoring of the dCO_2_ and pH signals dynamics is required. In the next section, we present a case study of such an experimental system where a precisely controlled photobioreactor is coupled with a membrane inlet mass spectrometer.

### 3.2 Experimental validation of the model predictions

**Figure 6:**
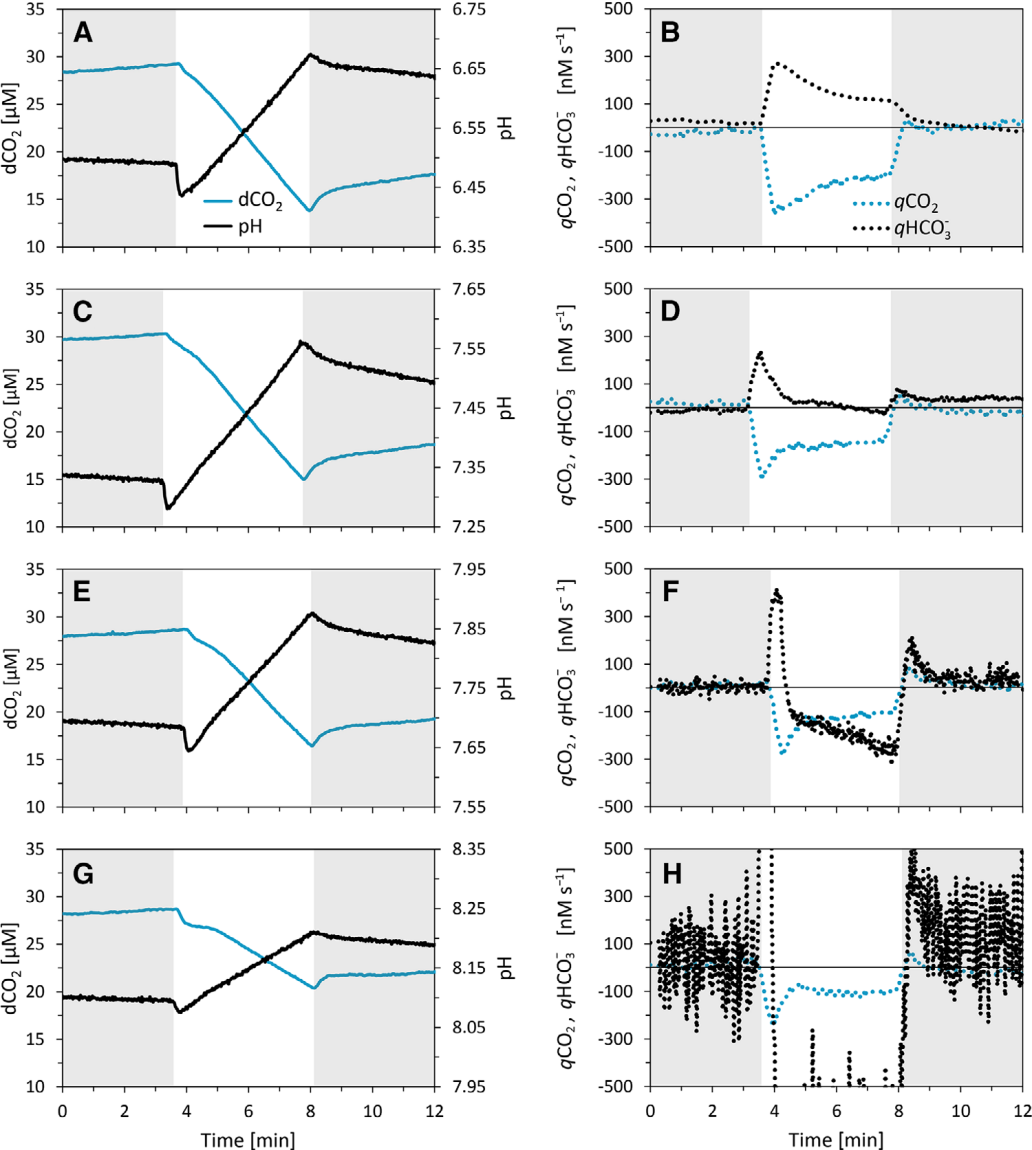
Experimental data of *Synechocystis* sp. PCC 6803 cultivated in non‐buffered BG‐11 medium during Dark‐Light‐Dark cycles: measurements of dCO_2_ (full blue lines) and pH signals (full black lines) signals (left panels) for initial pH 6.5 (panel A), 7.3 (panel C), 7.7 (panel E), and 8.1 (panel G). Four biological replicates with qualitatively identical results for each condition were collected; data from one representative experiment are shown. Identification of the exchange rates *q*CO_2_ (dotted blue lines, right panels) and *q* ${\mathrm{HCO}}_{3}^{-}$ (dotted black lines, right panels), identified from the measured data involving data differentiation and the assumption of *q*H^+^ = *q* ${\mathrm{HCO}}_{3}^{-}$ is represented in right panels for initial pH 6.5 (panel B), 7.3 (panel D), 7.7 (panel F), and 8.1 (panel H). Positive values in the right panels indicate dCO_2_ or ${\mathrm{HCO}}_{3}^{-}$ excretion, negative values indicate dCO_2_ or ${\mathrm{HCO}}_{3}^{-}$ uptake. For further experimental details, see Section 2

For the validation of our numerical simulations of the dCO_2_ and pH displacements under varying pH levels and buffer concentrations, we performed a series of Dark‐Light‐Dark experiments. High magnitudes of the dCO_2_ displacement under high buffer concentration are well documented in the literature 2, 21, 34, 36, 39, 40. Therefore, in this work, we focused on the experimental validation of ICC visibility in more natural, weakly buffered systems. To be consistent with the model predictions as summarized in Figure 3A–C, we performed a series of measurements under initial pH values of 6.5–8.1, and we monitored the dCO_2_ and pH dynamics in a *Synechocystis* sp. PCC 6803 culture during Dark‐Light‐Dark experiments. To be consistent with the simulations, the initial dCO_2_ concentration during the experiments was set to 30 µM. Additionally, all simulations shown in Figures 3, 4, 5 were performed to predict dCO_2_ and pH signals for “CO_2_ users” — organisms taking up a higher fraction of dCO_2_ compared to ${\mathrm{HCO}}_{3}^{-}$. The measured dCO_2_ dynamics in *Synechocystis* during our experiments also resembles dynamics typical for “CO_2_ users” 2. However, since we used low CO_2_ concentration (air), it is likely that all DIC transporters were active 41 and therefore *Synechocystis* was most likely able to uptake both dCO_2_ and ${\mathrm{HCO}}_{3}^{-}$. The results of the experiments are summarized in Figure 6 (left panels), and the results agree with the model predictions; under decreasing pH, the dCO_2_ displacement became less visible and, on the contrary, the pH displacement became more pronounced. In particular, the pH displacement was the most visible at pH 6.5 (ΔpH = 0.051, corresponding to ΔH^+^ = 40.9 nM), less visible at pH 7.3 (ΔpH = 0.042, corresponding with ΔH^+^ = 4.8 nM), even less visible at pH 7.7 (ΔpH = 0.036, corresponding with ΔH^+^ = 1.8 nM), and the least visible at pH 8.1 (ΔpH = 0.016, corresponding with ΔH^+^ = 0.3 nM). It is important to note that this agreement between simulations and experiments was achieved although the rates *q*CO_2_ and *q* ${\mathrm{HCO}}_{3}^{-}$ as identified from the experiments (summarized in the right panels of Figure 6 and further described in the next section 3.3) differed from rates considered in the simulations. There, we considered *q*CO_2_ and *q* ${\mathrm{HCO}}_{3}^{-}$ rates as constant, whereas in the experiments both rates were identified as dynamic (Figure 6, right panels). The net dO_2_ evolution (as a sum of all fluxes between *Synechocystis* cells and the aquatic environment under light) was almost identical for all pH levels (6.5–8.1), which indicates a negligible effect of the pH level on the light‐dependent photosynthetic reactions (see Supporting Information Figures S6 and S7).

### 3.3 Identifying exchange rates *q*CO_2_ and *q* ${\mathrm{HCO}}_{3}^{-}$ from experimental data

The mathematical model can also be used for identifying the exchange rates *q*CO_2_ and *q* ${\mathrm{HCO}}_{3}^{-}$ (and *q*H^+^) from the measured dCO_2_ and pH signals. Mathematically, the identification of three time series from two noisy time series is an ill‐posed problem. The development of a numerically stable identification algorithm is beyond the scope of this work. As a first step, we assume equal ${\mathrm{HCO}}_{3}^{-}$ and H^+^ exchange rates (and hence constant total alkalinity) and implement a simple algorithm that involves data differentiation; see Supporting Information Subsection 1.5. This approach works for sufficiently high S/N; in our case, for low pH values and low buffer concentrations. The measured data (Figure 6 left panels) were used for identifying the exchange rates *q*CO_2_ and *q* ${\mathrm{HCO}}_{3}^{-}$; the results are summarized in Figure 6, right panels. As it turned out, our simple algorithm is problematic only for a high pH of 8.1.

Inorganic carbon cycling (simultaneous dCO_2_ uptake and ${\mathrm{HCO}}_{3}^{-}$ excretion) was identified to be both qualitatively and quantitatively different for initial pH levels of 6.5–8.1. For initial pH 6.5 (Figure 6A–B), we identified ICC (with decreasing magnitude) throughout the whole light period and partially also during the dark period after light (Figure 6B). For initial pH 7.3 (Figure 6C–D), the exchange rate *q*CO_2_ was comparable to pH 6.5, whereas a significant exchange rate *q* ${\mathrm{HCO}}_{3}^{-}$ was present only for the first 90 s of the light phase (Figure 6D). For initial pH 7.7 (Figure 6E–F), massive ICC was identified for the first 20 s of and sole carbon fixation for the rest of the light phase. For initial pH 8.1 (Figure 6G–H), both *q*CO_2_ and *q* ${\mathrm{HCO}}_{3}^{-}$ were qualitatively identical as for pH 7.7, however, the *q* ${\mathrm{HCO}}_{3}^{-}$ magnitude was higher than for pH 7.7. Our results suggest that the magnitude of ICC can vary significantly over time and in fact, ICC may be present only at the beginning of the light phase.

## 4 DISCUSSION

### 4.1 Detection of inorganic carbon cycling

In the present work, we developed methods for a quantitative description of inorganic carbon cycling (ICC) between photosynthetic microorganisms and their aquatic environment. In particular, (i) we developed a mathematical model that quantitatively describes dCO_2_ and ${\mathrm{HCO}}_{3}^{-}$ fluxes (*q*CO_2_ and *q* ${\mathrm{HCO}}_{3}^{-}$) between cells and the environment, (ii) we predicted the visibility of ICC under various buffer concentrations and pH levels, and (iii) we experimentally validated the model predictions, using the combination of a precisely controlled photobioreactor and a high resolution membrane inlet mass spectrometer. In both simulations and experiments, we focused on slightly acidic to slightly alkaline pH range, since first, with pH increase, the pH displacement becomes hardly detectable even in non‐buffered systems due to (self‐) buffering capacity of the carbonate system, Figure 3A–C, Figure 6), and second, at pH < 6, dCO_2_ uptake can become significantly reduced or even inhibited 34.

Although ICC has been first described already in 1998 21, available methods used for ICC detection still rely on experimental evaluations of the dCO_2_ displacement at the beginning and at the end of the light phase (Figure 1). However, as we show in the simulations, in highly‐buffered cultivation media, such dCO_2_ displacement can be observed even in the absence of ICC (Figures 4, 5). On the other hand, in weakly buffered systems, dCO_2_ displacements are a clear sign of ICC. Quantitatively, we show that the magnitude of dCO_2_ displacements induced by ICC decreases between pH 8 and 7 (Figure 3A–C, Figure 6); and at pH ≤ 7, a dCO_2_ displacement cannot be detected any more (Figure 3A–C).

The model simulations further show that, with low buffer concentrations, it is possible to detect ICC from pH displacements (Figure 3A–C). Similarly to dCO_2_ displacements, also pH displacements result from abrupt changes of the exchange rates *q*CO_2_, *q* ${\mathrm{HCO}}_{3}^{-}$, and *q*H^+^. Thereby, the displacements occur with a certain delay corresponding to the “apparent” hydration rate constant *k*_1_(1+α); see Supporting Information Equation 4. For pH = 7, when the hydration rate *v* shows a very short delay compared to pH = 8 (Supporting Information Figure S3), simulations (Figure 3A) as well as experimental data (Figure 6) show only slightly delayed pH displacements. Since the “acceleration” parameter α decreases with increasing pH between 7–8 (Supporting Information Figure S3), the simulations predict that the corresponding delay of the pH displacement increases with increasing pH (in the range 7–8, Figure 3A–C). In the experiments, however, the exchange rates *q*CO_2_, *q* ${\mathrm{HCO}}_{3}^{-}$ (and *q*H^+^) identified from the experimental data (Figure 6) showed some delay (at the beginning of the light phase) under all tested pH levels, which indicates that the assumption of abrupt change used in the simulations may not be justified.

A further analysis showed that the magnitude of the pH displacement during ICC decreases with increasing pH in the range of 7–8 (Figure 3A–C, Figure 6), which is a consequence of the increased (self‐) buffering capacity of the carbonate system as well as water self‐dissociation; with pH increasing from 7 to 8, the concentrations of OH^−^ and ${\mathrm{HCO}}_{3}^{-}$ (in chemical equilibrium) increase 10 times, and protons that are excreted from the cells (pressumably together with ${\mathrm{HCO}}_{3}^{-}$) react with the both anions to a higher extent which leads to a reduced amount of “free” protons available for pH displacement.

As discussed above, the dCO_2_ displacement can be detected only when the dCO_2_ hydration rate constant *k*_1_ (part of the “apparent” hydration rate constant *k*_1_ (1+α)) is small enough — in particular, as shown in Supporting Information Figure S5, less than 1 s^−1^. In this work, we experimentally determined the value of *k*_1_ as 0.04 s^−1^ (Table 1), which has been also reported for sea water 42. Some photosynthetic microorganisms contain extracellular carbonic anhydrase (CA) 43, an enzyme that can increase *k*_1_ significantly. The turnover number *k*_cat_ of CA is usually reported in the range of 10^4^–10^6^ s^−1^, that is 10^6^–10^8^ times higher than *k*_1_. As shown in Supporting Information Figure S5, already a *k*_1_ value of 0.08 s^−1^ reduces the dCO_2_ displacement by one half, and at *k*_1_ ≥ 4, the dCO_2_ displacement cannot be detected anymore. For ICC detection in strains with external CA, it is therefore extremely important to ensure complete CA inhibition since even a minimal CA activity can prevent the ICC visibility.

### 4.2 Assumptions and limits of the presented carbonate chemistry model

Our mathematical model is based on several assumptions regarding carbonate chemistry such as fast equilibrium for reactions W, B, 1′, and 2^+^/2^−^ (see Figure 2 and Supporting Information Section 1.3 for further details). To preserve constant total alkalinity and charge balance, we also assume co‐transport of ${\mathrm{HCO}}_{3}^{-}$ and H^+^ (*q* ${\mathrm{HCO}}_{3}^{-}$ = *q*H^+^). The current understanding of proton efflux in cyanobacteria is fully consistent with this assumption. Even though current carbon concentrating mechanism models consider two ${\mathrm{HCO}}_{3}^{-}$ transporters (SbtA and BicA) that require extracellular Na^+^ which is exchanged with H^+^ by the antiporter NhaS3, the photosynthetic and respiratory models consider at least three complexes in the cytoplasmic membrane that all work as proton (efflux) pumps: NDH_1/2_, COX, and P‐ATPase 44. Indeed, proton efflux channels are present also in eukaryotic algae 24, 45. There is also direct evidence for ${\mathrm{HCO}}_{3}^{-}$ and H^+^ cotransport in some strains 46. Clearly, the assumption of equal integrals of *q* ${\mathrm{HCO}}_{3}^{-}$ and *q*H^+^ over time is reasonable; otherwise the cells would become acidified or basified over time. However, eventual fast fluctuations in both *q* ${\mathrm{HCO}}_{3}^{-}$ and *q*H^+^ (on the time scale of seconds) can result in temporal *q* ${\mathrm{HCO}}_{3}^{-}$ and *q*H^+^ disbalance. This can lead to (temporally) incorrect *q*CO_2_ and *q* ${\mathrm{HCO}}_{3}^{-}$ rates identification from experimental data (see the following section for further discussion).

The model further assumes that both kinetic and equilibrium constants (as summarized in Table 1) are known for the given physical and chemical properties of the aquatic environment such as temperature, salinity, total alkalinity, pH, or pressure. Finally, only three fluxes between cells and their aquatic environment are considered in the model (*q*CO_2_, *q* ${\mathrm{HCO}}_{3}^{-}$, and *q*H^+^, Figure 2), and fluxes of all other ions that can influence total alkalinity (such as Na^+^, K^+^, NO_3_^−^, NH_4_^+^ 47, 48) are neglected. Despite these limitations, the model provides a valuable contribution towards understanding and quantitative evaluation of inorganic carbon cycling.

### 4.3 Towards a reliable method for the identification of *q*CO_2_ and *q* ${\mathrm{HCO}}_{3}^{-}$

In addition to predicting the visibility of ICC, the model was further used to identify the exchange rates *q* ${\mathrm{HCO}}_{3}^{-}$ and *q*CO_2_ from the experimental data. We assessed ICC of various durations and magnitudes in the pH range 6.5–8.1 (Figure 6, right column). The *q*CO_2_ and *q* ${\mathrm{HCO}}_{3}^{-}$ identification algorithm involved data differentiation which turned out to be appropriate for data measured at pH ≤ 7.7. For pH = 8.1, this method amplified data noise in the pH signal, caused by an increasing (self‐)buffering capacity of carbonate chemistry and hence a higher S/N with increasing pH. Noise reduction in the identified exchange rate *q* ${\mathrm{HCO}}_{3}^{-}$ at higher pH can be achieved by advanced data fitting methods involving regularization techniques. However, without direct measurement of the ${\mathrm{HCO}}_{3}^{-}$ dynamics, the identification of *q*CO_2_ and *q* ${\mathrm{HCO}}_{3}^{-}$ fluxes may still depend on model assumptions such as *q* ${\mathrm{HCO}}_{3}^{-}$ = *q*H^+^ (see the previous section for further discussion).

Being aware of the model limitations outlined above, the exchange rates *q*CO_2_ and *q* ${\mathrm{HCO}}_{3}^{-}$ identified from experimental data can be interpreted only as rough guides of ICC in general. The first analysis of our experimental data showed that ICC could take place throughout the whole light period (Figure 6B), only partially during the light period (Figure 6D,F,H), and also partially in dark (Figure 6B), suggesting highly dynamic ICC and/or *q* ${\mathrm{HCO}}_{3}^{-}$ and *q*H^+^ co‐transport.

The amount of ATP “burned” during ICC by the cells can be significant since transport of ${\mathrm{HCO}}_{3}^{-}$ against the concentration gradient requires ATP 24, 25. Giving an oversimplified example based on our experimental data, *q* ${\mathrm{HCO}}_{3}^{-}$ as identified during the initial displacement at light was as high as 40 µM s^−1^ (excretion, Figure 6 right column) which corresponds with 20 amol cell^−1^ s^−1^. Assuming (for simplicity) that each ${\mathrm{HCO}}_{3}^{-}$ molecule requires 1/3 ATP to be excreted from the cell (only one out of three total ${\mathrm{HCO}}_{3}^{-}$ transporters require ATP), such a rate would burn 6.7 amol cell^−1^ s^−1^ ATP, that is, 35% of the ATP generated by photosynthetic light reactions: assuming 3 ATP produced per each O_2_ molecule, the dO_2_ production rate of 6.4 amol cell^−1^ s^−1^ (as derived from the data presented in Supporting Information Figures S6 and S7) would result in an ATP production rate of 19.2 amol cell^−1^ s^−1^ (and 6.7/19.2 = 35%). However, we note that a precise accounting of ATP (and reducing equivalents) production/consumption is tricky since the stoichiometry of both dO_2_:ATP production and ${\mathrm{HCO}}_{3}^{-}$ excretion:ATP consumption can vary considerably over time. In addition, under high blue light as used in our experiments, one can expect a strong cyclic electron flow around photosystem I (PSI) that can shift the ratio of ATP:NADPH production significantly 49. Considering higher ICC rates at the beginning of the light phase, compared to the rest of the light phase (Figure 6, right panels), our results suggest that ICC can serve as a temporal ATP‐burning mechanisms until other ATP‐consuming processes such as inorganic carbon fixation become activated.

The photosynthetic quotient in our experiments (PQ, net O_2_ release:CO_2_ fixation) was ranging between 5.7–0.3 in the pH range 6.5–8.1. Such values represent a higher PQ variation than reported previously for *Synechocystis*. However, in previous works 50, 51, 52 light of lower intensity and different wavelength was used. As mentioned above, in *Synechocystis* blue light preferentially affects PSI, resulting in cyclic electron flow 49.

The model analysis also showed that the negative slope of the dCO_2_ signal during the light phase does not reflect the true inorganic carbon fixation rate. In fact, it equals α/(1+α) times the true inorganic carbon fixation rate (*q*CO_2_ + *q* ${\mathrm{HCO}}_{3}^{-}$), see Supporting Information Equation 8. Thus, the dCO_2_ signal slope depends on the pH level (Figure 3), which was also confirmed experimentally (Figure 6 left column, Supporting Information Figure S7). The “apparent” rate is closest to the true rate around pH = pK1 = 6.3 (for large α), and the difference between the “apparent” and true inorganic carbon fixation rates increases with increasing pH and buffer concentrations — following the dependence of the “acceleration” parameter α on both pH and buffer concentration (see Supporting Information Equation 4 and Figure S2). As shown in Supporting Information Figure S7, the “apparent” and true inorganic carbon fixation rate, as identified from our experimental data, can differ by a factor of 10 under the highest tested pH 8.1.

A reliable method for the dynamic identification of both exchange rates *q*CO_2_ and *q* ${\mathrm{HCO}}_{3}^{-}$, and thus for the assessment of ICC is still missing. This work summarizes the complexity and limitations of ICC detection related to the effects of carbonate chemistry and to the current state of the art of the experimental techniques. Until a method for the dynamic and quantitative measurement of ${\mathrm{HCO}}_{3}^{-}$ concentrations will be available, both *q*CO_2_ and *q* ${\mathrm{HCO}}_{3}^{-}$ quantification will depend on a mathematical analysis involving extra assumptions. In this work, we outlined future developments of both experimental and modeling methods as necessary for a reliable ICC detection.

## OPEN DATA

Source data for Figure 6 are available online here: https://identifiers.org/ecyano.experiment:31. Mathematical model is available online here: https://identifiers.org/ecyano.model:46.

## CONFLICT OF INTEREST

The authors have declared no conflict of interest.

## Supporting information

Supporting Information
