# Supplementary material for: Towards a quantitative assessment of inorganic carbon cycling in photosynthetic microorganisms
Source: Eng Life Sci. 2019 Oct 31;19(12):955–67. doi: 10.1002/elsc.201900061 (PMC6999069; doi:10.1002/elsc.201900061)
Supplement: Supplementary file 1 — Supporting Information [file ELSC-19-955-s001.pdf]

Supporting Information

Towards a quantitative assessment of inorganic carbon cycling in photosynthetic microorganisms

Stefan Müller, Tomáš Zavřel, Jan Červený

1 Mathematical model

1.1 Notation

Variables:

$[CO_2]$	...	concentration of dissolved CO_2
$[HCO_3^-]$	...	concentration of HCO_3^-
$[H^+]$	...	concentration of H^+
CP	...	carbonate pool
TA	...	total alkalinity
qCO_2	...	exchange rate of CO_2
$qHCO_3^-$	...	exchange rate of HCO_3^-
qH^+	...	exchange rate of H^+
v	...	net hydration rate

Constants:

k_1	...	hydration rate “constant”
K_1	...	equilibrium constant of $CO_2 + H_2O \rightleftharpoons HCO_3^- + H^+$
K_2	...	equilibrium constant of $HCO_3^- \rightleftharpoons CO_3^{2-} + H^+$
K_W	...	water self-dissociation constant
K_B	...	equilibrium constant of buffer
c_B^{tot}	...	concentration of buffer

1.2 Carbonate chemistry

Carbonate chemistry, water self-dissociation, and buffer:

$$
\begin{array}{lcll}
1' & : & CO_2 + H_2O & \rightleftharpoons H_2CO_3 \\
1'' & : & H_2CO_3 & \rightleftharpoons HCO_3^- + H^+ \\
1^+ & : & CO_2 + H_2O & \rightleftharpoons HCO_3^- + H^+ \\
1^- & : & CO_2 + OH^- & \rightleftharpoons HCO_3^- \\
2^+ & : & HCO_3^- & \rightleftharpoons CO_3^{2-} + H^+ \\
2^- & : & HCO_3^- + OH^- & \rightleftharpoons CO_3^{2-} + H_2O \\
W & : & H_2O & \rightleftharpoons H^+ + OH^- \\
B & : & HA & \rightleftharpoons H^+ + A^-
\end{array}
$$

1.3 Model derivation

i. We assume equilibrium for the fast reactions W and B,

$$K_W = [H^+][OH^-], \quad K_B = \frac{[H^+][A^-]}{[HA]},$$

and use mass conservation,

$$[HA] + [A^-] = c_B^{tot},$$

to eliminate the species $OH^-$and HA, A^-. In particular, we obtain:

$$[A^-] = c_B^{tot} \frac{K_B}{K_B + [H^+]}.$$

ii. We assume equilibrium for the fast reactions $1''$ and $2^+/2^-$,

$$K_{1''} = \frac{[HCO_3^-][H^+]}{[H_2CO_3]}, \quad K_2 = \frac{[CO_3^{2-}][H^+]}{[HCO_3^-]}$$

with

$$K_2 := K_{2^+} = K_{2^-} \cdot K_W,$$

to eliminate the species H_2CO_3 and CO_3^{2-}.

iii. We introduce a carbonate pool which is unchanged by the fast reactions $1''$ and $2^+/2^-$:

$$\begin{aligned} CP &:= [H_2CO_3] + [HCO_3^-] + [CO_3^{2-}] \\ &= [HCO_3^-] \left(1 + \frac{[H^+]}{K_{1''}} + \frac{K_2}{[H^+]}\right) \\ &= [HCO_3^-] \left(1 + \frac{K_2}{[H^+]}\right). \end{aligned}$$

In the last step, we assume $\frac{[H^+]}{K_{1''}} \ll 1$. For example, $\frac{[H^+]}{K_{1''}} < 10^{-2}$ corresponds to $pH > pK_{1''} + 2 \approx 3.6 + 2 = 5.6$. In our experiments, we consider pH values from 6.5 to 8.1, and the assumption is well justified.

iv. We compute the net rate of the reaction between CO_2 and the carbonate pool CP, determined by the slow reactions $1'$ and $1^+/1^-$:

$$\begin{aligned} v = &+ \overrightarrow{k_{1'}}\,[CO_2] - \overleftarrow{k_{1'}}\,[H_2CO_3] \\ &+ \overrightarrow{k_{1^+}}\,[CO_2] - \overleftarrow{k_{1^+}}\,[HCO_3^-][H^+] \\ &+ \overrightarrow{k_{1^-}}\,[OH^-][CO_2] - \overleftarrow{k_{1^-}}\,[HCO_3^-] \\ = &+ \left(\overrightarrow{k_{1'}} + \overrightarrow{k_{1^+}} + \overrightarrow{k_{1^-}}\,\frac{K_W}{[H^+]}\right)[CO_2] - \left(\frac{\overleftarrow{k_{1'}}}{K_{1''}} + \overleftarrow{k_{1^+}} + \frac{\overleftarrow{k_{1^-}}}{[H^+]}\right)[HCO_3^-][H^+] \\ = &+ k_1\,[CO_2] - \left(\frac{\overrightarrow{k_{1'}}}{K_{1'}K_{1''}} + \frac{\overrightarrow{k_{1^+}}}{K_{1^+}} + \frac{\overrightarrow{k_{1^-}}}{K_{1^-}[H^+]}\right)[HCO_3^-][H^+] \\ = &+ k_1\,[CO_2] - \left(\overrightarrow{k_{1'}} + \overrightarrow{k_{1^+}} + \overrightarrow{k_{1^-}}\,\frac{K_W}{[H^+]}\right)[HCO_3^-]\,\frac{[H^+]}{K_1} \\ = &+ k_1\left([CO_2] - [HCO_3^-]\,\frac{[H^+]}{K_1}\right) \end{aligned}$$

Thereby, we

- assume equilibrium for the fast reactions W and $1''$ (cf. i and ii),
- use the equality of the equilibrium constants for the three mechanisms $1' + 1''$, 1^+, and $1^- + \mathrm{W}$,

$$
\begin{aligned}
K_1 &= K_{1'} \cdot K_{1''} = \frac{k_{1'}^{\rightharpoonup}}{k_{1'}^{\leftharpoondown}} K_{1''} \\
&= K_{1^+} = \frac{k_{1^+}^{\rightharpoonup}}{k_{1^+}^{\leftharpoondown}} \\
&= K_{1^-} \cdot K_\mathrm{W} = \frac{k_{1^-}^{\rightharpoonup}}{k_{1^-}^{\leftharpoondown}} K_\mathrm{W},
\end{aligned}
$$

- define the hydration rate "constant"

$$
k_1 = k_{1'}^{\rightharpoonup} + k_{1^+}^{\rightharpoonup} + k_{1^-}^{\rightharpoonup} \frac{K_\mathrm{W}}{[\mathrm{H}^+]}. \tag{1}
$$

v. Finally, we introduce total alkalinity which is unchanged by carbonate chemistry, water self-dissociation, and buffering (due to charge balancing in chemical reactions),

$$
\begin{aligned}
TA &= +\,[\mathrm{HCO_3^-}] + 2\,[\mathrm{CO_3^{2-}}] - [\mathrm{H}^+] + [\mathrm{OH}^-] + [\mathrm{A}^-] \\
&= +\,[\mathrm{HCO_3^-}] \left(1 + \frac{2\,K_2}{[\mathrm{H}^+]}\right) - [\mathrm{H}^+] + \frac{K_\mathrm{W}}{[\mathrm{H}^+]} + c_\mathrm{B}^{tot} \frac{K_\mathrm{B}}{K_\mathrm{B} + [\mathrm{H}^+]}.
\end{aligned}
$$

Total alkalinity is only changed by the culture through $\mathrm{HCO_3^-}$ and H^+ exchange.

To summarize, we assume equilibrium for the fast reactions $1''$, $2^+/2^-$, W and B (and eliminate 5 out of 8 species), we introduce the carbonate pool CP and total alkalinity TA (and end up with $5 = 8 - 5 + 2$ species), and we define the net hydration rate, determined by the slow reactions $1'$ and $1^+/1^-$,

$$
v = k_1 \left([\mathrm{CO_2}] - [\mathrm{HCO_3^-}] \frac{[\mathrm{H}^+]}{K_1} \right). \tag{2}
$$

Given the exchange rates $q\mathrm{CO_2}$, $q\mathrm{HCO_3^-}$, $q\mathrm{H}^+$, we obtain 5 equations (3 ordinary differential equations and 2 algebraic equations) for the 5 variables $[\mathrm{CO_2}]$, $[\mathrm{HCO_3^-}]$, $[\mathrm{H}^+]$, CP, TA:

$$
\frac{\mathrm{d}[\mathrm{CO_2}]}{\mathrm{d}t} = -\,v + q\mathrm{CO_2} \tag{3a}
$$

$$
\frac{\mathrm{d}\,CP}{\mathrm{d}t} = +\,v + q\mathrm{HCO_3^-} \tag{3b}
$$

$$
\frac{\mathrm{d}\,TA}{\mathrm{d}t} = +\,q\mathrm{HCO_3^-} - q\mathrm{H}^+ \tag{3c}
$$

$$
CP = [\mathrm{HCO_3^-}] \left(1 + \frac{K_2}{[\mathrm{H}^+]}\right) \tag{3d}
$$

$$
TA = [\mathrm{HCO_3^-}] \left(1 + \frac{2\,K_2}{[\mathrm{H}^+]}\right) - [\mathrm{H}^+] + \frac{K_\mathrm{W}}{[\mathrm{H}^+]} + c_\mathrm{B}^{tot} \frac{1}{1 + \frac{[\mathrm{H}^+]}{K_\mathrm{B}}} \tag{3e}
$$

Note that the ordinary differential equations (3abc) take this very simple form because of the introduction of the carbonate pool *CP* (and the use of total alkalinity *TA*). In particular, the effect of hydration (v) and exchange ($q\mathrm{CO_2}$, $q\mathrm{HCO_3^-}$, $q\mathrm{H^+}$) is very transparent.

Solving the DAE system (3) requires the following values:

– constants: k_1, K_1, K_2, K_W, K_B, c_B^{tot}
(for given medium at temperature T and ionic strength I)

– initial conditions: either $[\mathrm{CO_2}]$, CP, TA or e.g. $[\mathrm{CO_2}]$, $[\mathrm{HCO_3^-}]$, $[\mathrm{H^+}]$

For numerical solutions, we use *Wolfram Mathematica 10*, in particular, the function *NDSolve* with default options.

1.4 Model analysis

In order to explain the (fast) dynamics following the change of light conditions (dark/light or light/dark), we further analyze hydration.

For given exchange rates $q\mathrm{CO_2}$, $q\mathrm{HCO_3^-}$, $q\mathrm{H^+}$ and hydration rate constant k_1, the hydration rate v is determined by the following ODE:

$$\begin{aligned}
\frac{1}{k_1}\frac{\mathrm{d}v}{\mathrm{d}t} &= \frac{\mathrm{d}}{\mathrm{d}t}\left([\mathrm{CO_2}] - \frac{[\mathrm{HCO_3^-}]\,[\mathrm{H^+}]}{K_1}\right) \qquad (4)\\
&= \frac{\mathrm{d}[\mathrm{CO_2}]}{\mathrm{d}t} - \frac{[\mathrm{H^+}]}{K_1}\frac{\mathrm{d}[\mathrm{HCO_3^-}]}{\mathrm{d}t} - \frac{[\mathrm{HCO_3^-}]}{K_1}\frac{\mathrm{d}[\mathrm{H^+}]}{\mathrm{d}t}\\
&= \frac{\mathrm{d}[\mathrm{CO_2}]}{\mathrm{d}t} - \alpha\,\frac{\mathrm{d}CP}{\mathrm{d}t} + \beta\,\frac{\mathrm{d}TA}{\mathrm{d}t} \quad \text{(using Eqns. (3de))}\\
&= -\,v + q\mathrm{CO_2} - \alpha\left(v + q\mathrm{HCO_3^-}\right) + \beta\left(q\mathrm{HCO_3^-} - q\mathrm{H^+}\right) \quad \text{(using Eqns. (3)abc)}\\
&= -\,(1+\alpha)\,v + q\mathrm{CO_2} - \alpha\,q\mathrm{HCO_3^-} + \beta\left(q\mathrm{HCO_3^-} - q\mathrm{H^+}\right)
\end{aligned}$$

Thereby, we introduce dimensionless variables α and β, which depend on $\mathrm{HCO_3^-}$, $\mathrm{H^+}$, and buffer concentrations in a complicated way:

$$\alpha = \frac{[\mathrm{H^+}]^2\Big([\mathrm{H^+}]\Big([\mathrm{H^+}]\Big(K_\mathrm{B}(c_\mathrm{B}^{tot}+K_\mathrm{B})+[\mathrm{H^+}]([\mathrm{H^+}]+2K_\mathrm{B})+K_\mathrm{W}\Big)+2K_\mathrm{B}K_\mathrm{W}\Big)+[\mathrm{HCO_3^-}]([\mathrm{H^+}]+4K_2)([\mathrm{H^+}]+K_\mathrm{B})^2+K_\mathrm{B}^2K_\mathrm{W}\Big)}{K_1([\mathrm{H^+}]+K_2)\Big([\mathrm{H^+}]\Big([\mathrm{H^+}]\Big(K_\mathrm{B}(c_\mathrm{B}^{tot}+K_\mathrm{B})+2K_\mathrm{B}[\mathrm{H^+}]+[\mathrm{H^+}]^2+K_\mathrm{W}\Big)+2K_\mathrm{B}K_\mathrm{W}\Big)+K_\mathrm{B}^2K_\mathrm{W}\Big)+K_1K_2[\mathrm{H^+}][\mathrm{HCO_3^-}]([\mathrm{H^+}]+K_\mathrm{B})^2}$$

$$\beta = \frac{[\mathrm{H^+}]^2[\mathrm{HCO_3^-}]([\mathrm{H^+}]+2K_2)([\mathrm{H^+}]+K_\mathrm{B})^2}{K_1([\mathrm{H^+}]+K_2)\Big([\mathrm{H^+}]\Big([\mathrm{H^+}]\Big(K_\mathrm{B}(c_\mathrm{B}^{tot}+K_\mathrm{B})+2K_\mathrm{B}[\mathrm{H^+}]+[\mathrm{H^+}]^2+K_\mathrm{W}\Big)+2K_\mathrm{B}K_\mathrm{W}\Big)+K_\mathrm{B}^2K_\mathrm{W}\Big)+K_1K_2[\mathrm{H^+}][\mathrm{HCO_3^-}]([\mathrm{H^+}]+K_\mathrm{B})^2}$$

See also Figure S2. The variables α and β are obtained by time differentiation of Eqns. (3de) and by solving the resulting linear system for the time derivatives $\frac{\mathrm{d}[\mathrm{HCO_3^-}]}{\mathrm{d}t}$, $\frac{\mathrm{d}[\mathrm{H^+}]}{\mathrm{d}t}$ in terms of $\frac{\mathrm{d}CP}{\mathrm{d}t}$, $\frac{\mathrm{d}TA}{\mathrm{d}t}$.

As a result, we obtain a nonhomogeneous linear ODE for v with the "apparent" hydration rate constant $k_1(1+\alpha)$. In situations where $\alpha \gg 1$, the dynamics following an abrupt change of light conditions (dark/light or light/dark) is much faster than expected from the hydration rate constant k_1.

In the main text, we often assume $q\mathrm{HCO_3^-} = q\mathrm{H^+}$ resulting from charge balance and implying $TA = const.$ For notational simplicity, we also make this assumption here, and Eqn. (4) becomes

$$\frac{1}{k_1}\frac{\mathrm{d}v}{\mathrm{d}t} = -\,(1+\alpha)\,v + q\mathrm{CO_2} - \alpha\,q\mathrm{HCO_3^-}. \qquad (5)$$

Abrupt light change

We consider an abrupt change in light conditions at $t = 0$ (either dark/light or light/dark), in particular, a step change in the exchange rates $q\mathrm{CO_2}$, $q\mathrm{HCO_3^-}$, $q\mathrm{H^+}$. We denote the phase $t < 0$ by $\ominus$ and the phase $t \geq 0$ by $\oplus$. Assuming $\alpha \approx \mathit{const}$, Eqn. (5) can be solved for $t \geq 0$ as

$$v(t) = (\bar{v}^{\ominus} - \bar{v}^{\oplus})\, \mathrm{e}^{-(1+\alpha)k_1 t} + \bar{v}^{\oplus}, \tag{6}$$

where

$$\bar{v} = \frac{q\mathrm{CO_2} - \alpha\, q\mathrm{HCO_3^-}}{1+\alpha}$$

is the quasi steady-state value of v; in particular, $\bar{v}^{\ominus}$ is the quasi steady-state value of v for $t < 0$, that is, $\bar{v}^{\ominus} = (q\mathrm{CO_2}^{\ominus} - \alpha\, q\mathrm{HCO_3^-}^{\ominus})/(1+\alpha)$; analogously, $\bar{v}^{\oplus}$ for $t \geq 0$. The hydration rate v starts from $\bar{v}^{\ominus}$ at $t = 0$ and approaches $\bar{v}^{\oplus}$ within $t \approx \frac{1}{(1+\alpha)\, k_1}$, leading to a "displacement" of

$$\Delta v = \bar{v}^{\oplus} - \bar{v}^{\ominus}.$$

Using the solution for v, the ODE for $[\mathrm{CO_2}]$, Eqn. (3a), can be solved for $t \geq 0$ as

$$\begin{aligned} [\mathrm{CO_2}](t) - [\mathrm{CO_2}](0) &= -\int_0^t v \,\mathrm{d}t' + q\mathrm{CO_2}\, t \\ &= \frac{\bar{v}^{\ominus} - \bar{v}^{\oplus}}{(1+\alpha)\, k_1}\, \mathrm{e}^{-(1+\alpha)k_1 t'} \Big|_0^t + \left(-\bar{v}^{\oplus} + q\mathrm{CO_2}\right) t \\ &= \frac{\Delta v}{(1+\alpha)\, k_1} \left(1 - \mathrm{e}^{-(1+\alpha)k_1 t}\right) + k\, t, \end{aligned} \tag{7}$$

leading to a "displacement" of

$$\Delta[\mathrm{CO_2}] = \frac{\Delta v}{(1+\alpha)\, k_1},$$

followed by a slow linear change with a slope of

$$k = \frac{\alpha}{1+\alpha} \left(q\mathrm{CO_2} + q\mathrm{HCO_3^-}\right). \tag{8}$$

The simplifying assumption $\alpha \approx \mathit{const}$ yields analytical solutions that are in good agreement with numerical solutions of the DAE system (3).

1.5 Identification of exchange rates

From measured concentrations $[\mathrm{CO_2}](t)$ and $[\mathrm{H^+}](t)$, we identify the exchange rates $q\mathrm{CO_2}(t)$ and $q\mathrm{HCO_3^-}(t)$.

Again, we assume $q\mathrm{HCO_3^-} = q\mathrm{H^+}$ resulting from charge balance and implying $\mathit{TA} = \mathit{const}$. First, Eqn. (3e) yields $[\mathrm{HCO_3^-}]$ as a function of $[\mathrm{H^+}]$,

$$[\mathrm{HCO_3^-}] = f([\mathrm{H^+}]) = \frac{\mathit{TA} + [\mathrm{H^+}] - \frac{K_\mathrm{W}}{[\mathrm{H^+}]} - c_\mathrm{B}^{tot} \frac{1}{1+\frac{[\mathrm{H^+}]}{K_\mathrm{B}}}}{1 + \frac{2\,K_2}{[\mathrm{H^+}]}}.$$

Next, insertion in Eqn. (3d) and time differentiation yields

$$\frac{\mathrm{d}CP}{\mathrm{d}t} = g([\mathrm{H}^+])\,\frac{\mathrm{d}[\mathrm{H}^+]}{\mathrm{d}t},$$

where g depends on $[\mathrm{H}^+]$ in a complicated way:

$$g = \frac{2K_2^2K_\mathrm{B}^2K_\mathrm{W} + g_1[\mathrm{H}^+] + g_2[\mathrm{H}^+]^2 + g_3[\mathrm{H}^+]^3 + g_4[\mathrm{H}^+]^4 + g_5[\mathrm{H}^+]^5 + [\mathrm{H}^+]^6}{[\mathrm{H}^+]^2(2K_2 + [\mathrm{H}^+])^2(K_\mathrm{B} + [\mathrm{H}^+])^2}$$

with

$$\begin{aligned}
g_1 &= 2K_2K_\mathrm{B}(2K_2 + K_\mathrm{B})K_\mathrm{W} \\
g_2 &= c_\mathrm{B}^{tot}K_2(2K_2 - K_\mathrm{B})K_\mathrm{B} + K_\mathrm{B}^2K_\mathrm{W} + 2K_2^2(K_\mathrm{B}^2 + K_\mathrm{W}) + K_2K_\mathrm{B}(4K_\mathrm{W} + K_\mathrm{B}\,TA) \\
g_3 &= 2(c_\mathrm{B}^{tot}K_2K_\mathrm{B} + (K_2 + K_\mathrm{B})(2K_2K_\mathrm{B} + K_\mathrm{W}) + K_2K_\mathrm{B}\,TA) \\
g_4 &= 2K_2^2 + K_\mathrm{B}(c_\mathrm{B}^{tot} + K_\mathrm{B}) + K_\mathrm{W} + K_2(8K_\mathrm{B} + TA) \\
g_5 &= 4K_2 + 2K_\mathrm{B}
\end{aligned}$$

Finally, Eqn. (2) becomes

$$v = k_1\left([\mathrm{CO_2}] - f([\mathrm{H}^+])\,\frac{[\mathrm{H}^+]}{K_1}\right) \tag{9}$$

and $q\mathrm{CO_2}$ and $q\mathrm{HCO_3^-}$ can be identified from $[\mathrm{CO_2}]$ and $[\mathrm{H}^+]$ via Eqns. (3ab):

$$q\mathrm{CO_2} = +\,v + \frac{\mathrm{d}[\mathrm{CO_2}]}{\mathrm{d}t} \tag{10a}$$

$$q\mathrm{HCO_3^-} = -\,v + g([\mathrm{H}^+])\,\frac{\mathrm{d}[\mathrm{H}^+]}{\mathrm{d}t} \tag{10b}$$

The identification of exchange rates involves the time differentiation of measured concentrations which may lead to noise amplification.

1.6 Model simulations

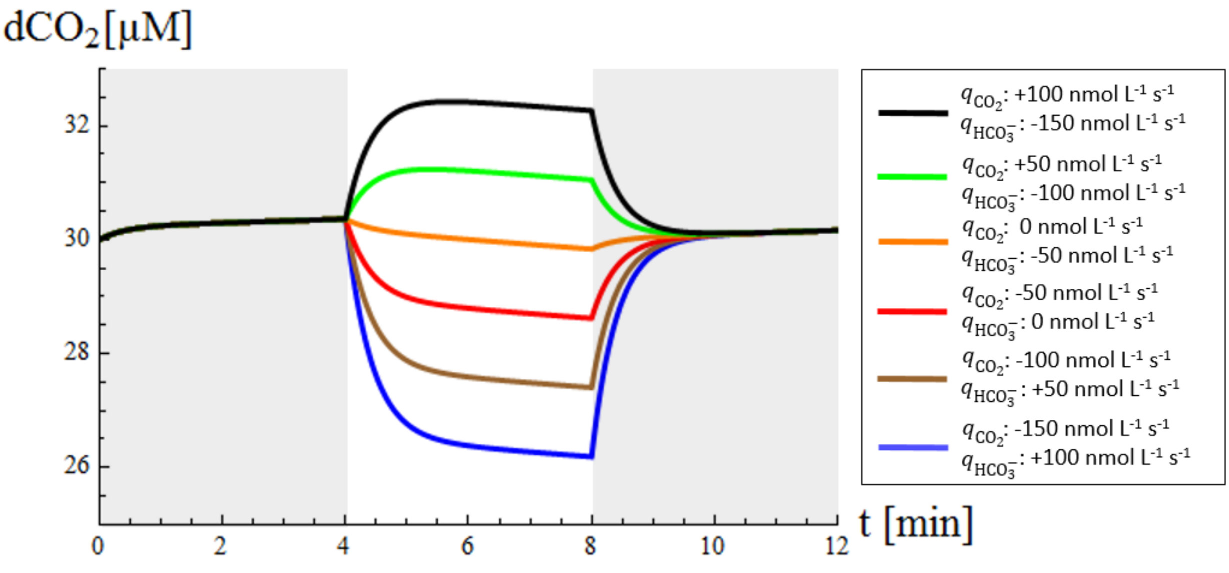

Figure S1 Visibility of dCO_2 displacements during Dark-Light-Dark experiments with qCO2 and $qHCO_3^-$ assumed between -150 - +100 nmol L-1 s-1. Initial dCO2 concentration set to 30 μM, initial pH value set to 8.0, and buffer (HEPES) concentration set to 17 mM. Exchange rates rate in dark set to 10 nmol L^{-1} s^{-1} for both qCO_2 and $qHCO_3^-$. Exchange rates qCO_2 and $qHCO_3^-$ at light set to: -150 nmol L^{-1} s^{-1} and +100 nmol L^{-1} s^{-1} (blue line), -100 nmol L^{-1} s^{-1} and +50 nmol L^{-1} s^{-1} (brown line), -50 nmol L^{-1} s^{-1} and 0 nmol L^{-1} s^{-1} (red line), 0 nmol L^{-1} s^{-1} and -50 nmol L^{-1} s^{-1} (orange line), +50 nmol L^{-1} s^{-1} and -100 nmol L^{-1} s^{-1} (green line) and +100 nmol L^{-1} s^{-1} and -150 nmol L^{-1} s^{-1} (black line). Grey rectangles: dark phases; white rectangle: light phase. Blue, brown, red, and orange lines resemble dCO_2 dynamics typical for 'CO_2 users'; green and black lines resemble dCO_2 dynamics typical for 'HCO_3^- users'.

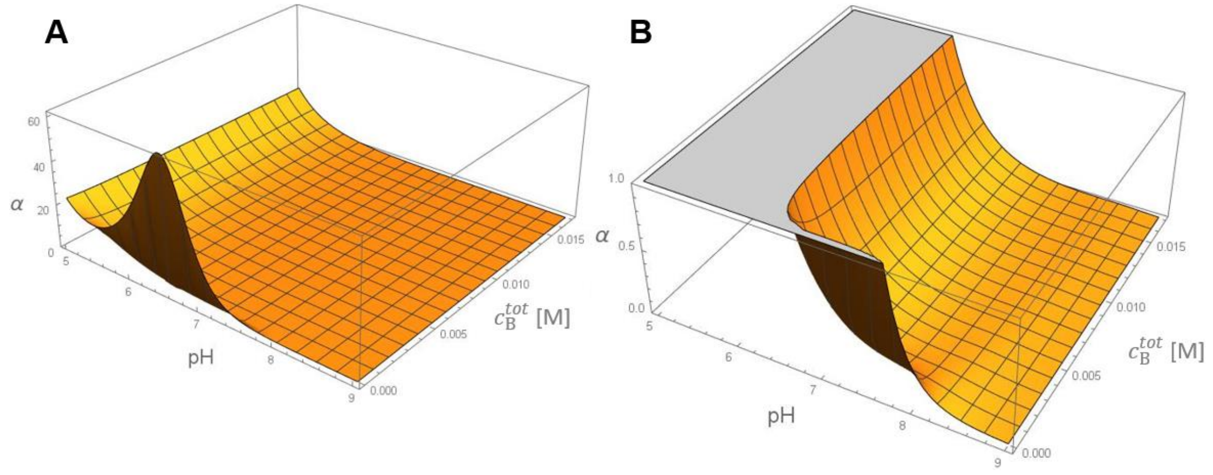

Figure S2 Dependence of the parameter α (defined after Equation (4)) on pH and buffer (HEPES) concentration c_B^{tot}. Panel A shows the entire range of the parameter α (for given pH and c_B^{tot}), panel B zooms in to α ≤ 1.

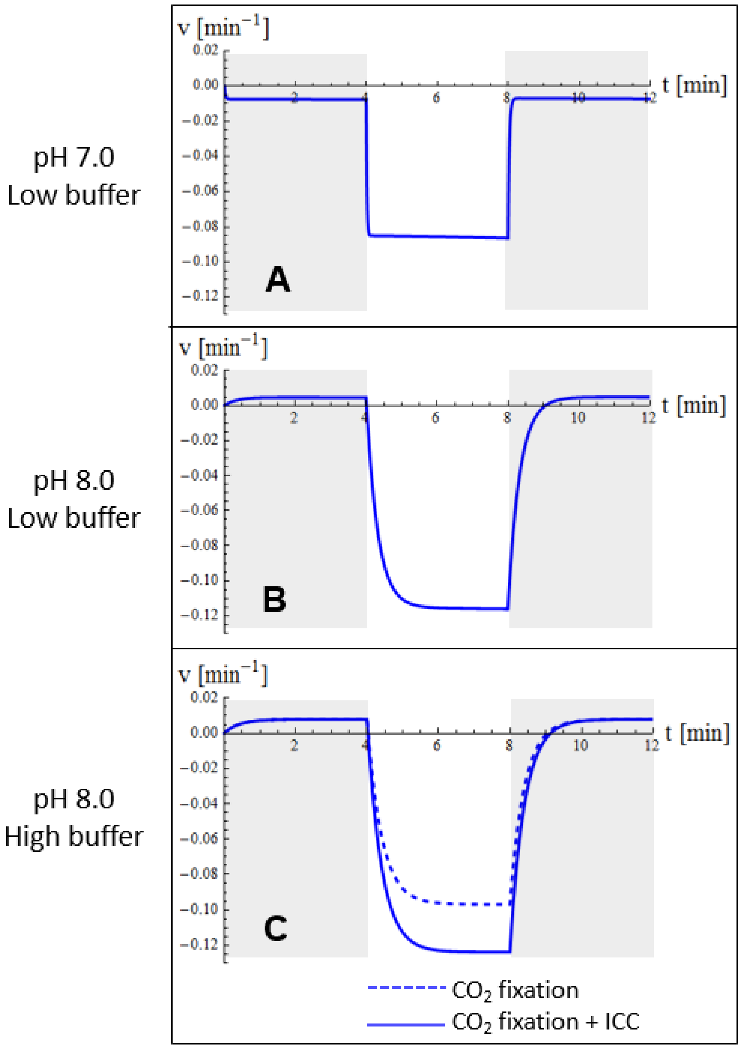

Figure S3 Dependence of the dCO_2 hydration rate *v* (see Equation (1) for further details) during Dark-Light-Dark experiments on pH (panels A, B) and buffer concentration (panels B, C). Initial dCO_2 concentration set to 30 µM, and buffer (HEPES) concentration set to 1 µM (panels A, B) or 17 mM (panel C). Exchange rates in dark set to +10 nmol L^{-1} s^{-1} for both CO_2 and HCO_3^- To simulate inorganic carbon cycling (ICC, full lines), exchange rates at light were set to -150 nmol L^{-1} s^{-1} for qCO_2 (uptake) and +100 nmol L^{-1} s^{-1} for $qHCO_3^-$ (excretion). To simulate carbon uptake in the absence of ICC (dashed line), exchange rates at light were set to -50 nmol L^{-1} s^{-1} for qCO_2 (uptake) and 0 nmol L^{-1} s^{-1} for $qHCO_3^-$. Grey rectangles: dark phases; white rectangles: light phases.

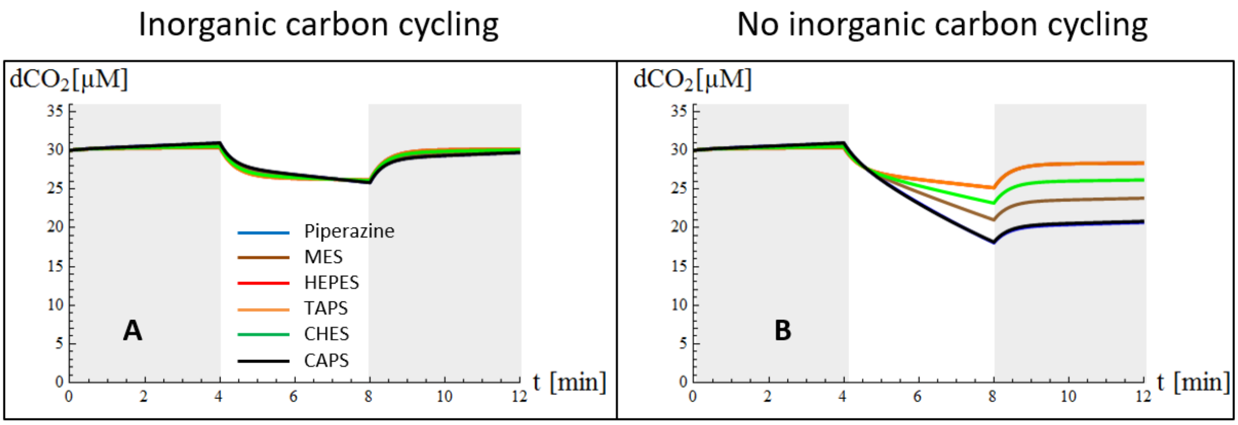

Figure S4 Dependence of dCO_2 displacement on pKa of different buffers during Dark-Light-Dark experiments, for CO_2 uptake complemented with inorganic carbon cycling (ICC, panel A) and CO_2 uptake without ICC (panel B). Initial dCO_2 concentration set to 30 μM, pH set to 8.0, and buffer concentrations set to 17 mM. List of buffers: piperazine (pKa = 5.59; blue lines), MES (pKa = 6.18; brown lines), HEPES (pKa = 7.55; red lines), TAPS (pKa = 8.45; orange lines), CHES (pKa = 9.44; green lines) and CAPS (pKa = 10.51, black lines). Exchange rates in dark set to +10 nmol L^{-1} s^{-1} for both qCO_2 and $qHCO_3^-$. To simulate ICC, exchange rates at light were set to -150 nmol L^{-1} s^{-1} for qCO_2 (uptake) and +100 nmol L^{-1} s^{-1} for $qHCO_3^-$ (excretion). To simulate absence of ICC, exchange rates at light were set to -125 nmol L^{-1} s^{-1} for qCO_2 and -200 nmol L^{-1} s^{-1} for $qHCO_3^-$. Grey rectangles: dark phases; white rectangles: light phases. (Note: blue and black lines represent almost identical dynamics.)

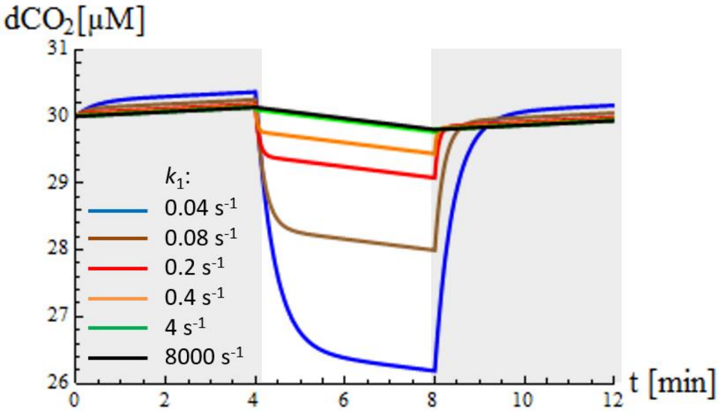

Figure S5 Dependence of dCO_2 displacement on dCO2 hydration rate constant k1 in the presence of inorganic carbon cycling during Dark-Light-Dark experiments. Initial dCO_2 concentration set to 30 μM, pH set to 8.0, and buffer concentration (HEPES) set to 17 mM. Exchange rates in dark set to +10 nmol L^{-1} s^{-1} for both qCO_2 and $qHCO_3^-$. Exchange rates at light set to -150 nmol L^{-1} s^{-1} for qCO_2 (uptake) and +100 nmol L^{-1} s^{-1} for $qHCO_3^-$ (excretion). Rate constant k_1 set to 0.04 s^{-1} (blue line), 0.08 s^{-1} (brown line), 0.2 s^{-1} (red line), 0.4 s^{-1} (orange line), 4 s^{-1} (green line), and 8 000 s^{-1} (black line). Grey rectangles: dark phases; white rectangle: light phase. (Note: green and black lines represent almost identical dynamics.)

2 Experimental data

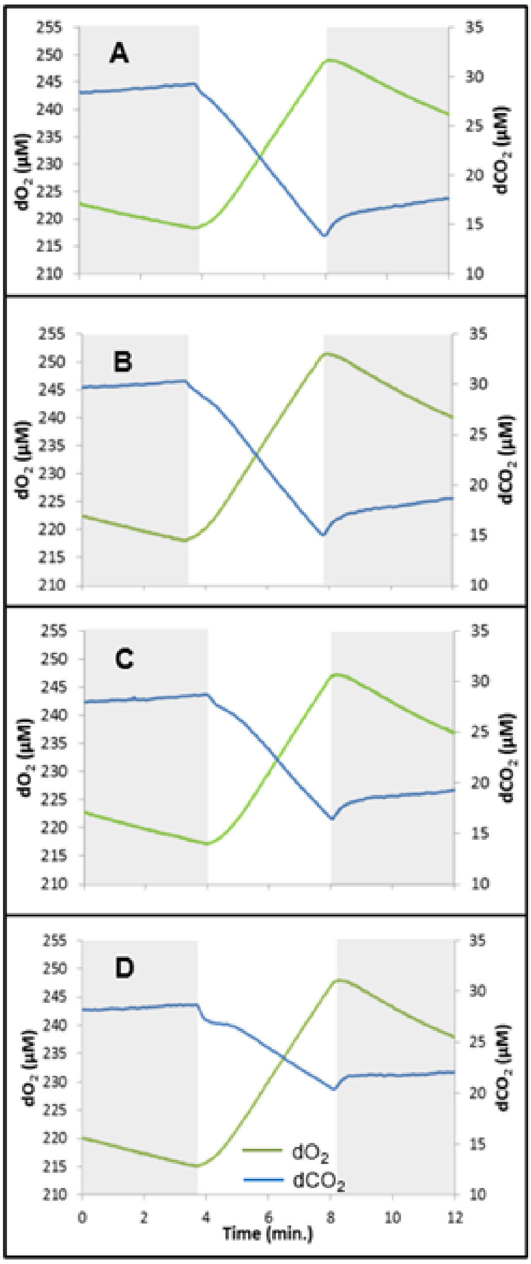

Figure S6 Experimental data of dCO_2 and dO_2 dynamics during Dark-Light-Dark experiments with *Synechocystis* sp. PCC 6803 under initial pH of 6.5 (panel A), 7.3 (panel B), 7.7 (panel C), and 8.1 (panel D). Both dCO_2 and dO_2 dynamics measured by membrane inlet mass spectrometer (MIMS); *Synechocystis* cultivated in a photobioreactor. Experiments performed in four biological replicates with qualitatively identical results, data from representative experiments shown. Grey rectangles: dark phases; white rectangles: light phases.

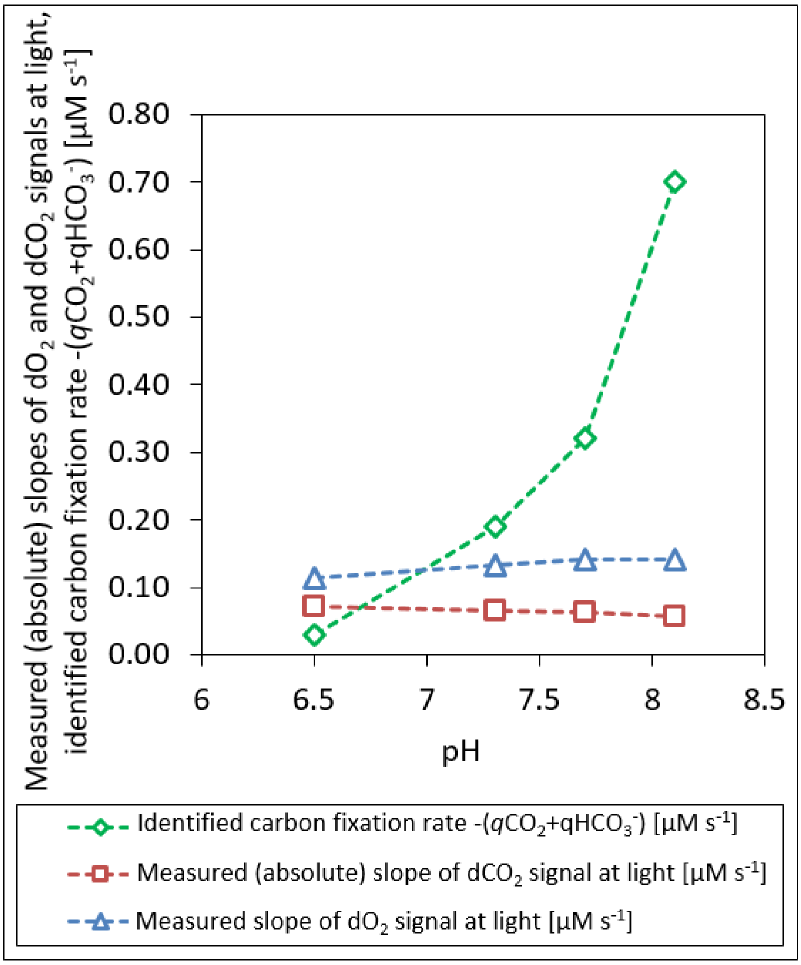

Figure S7 Comparison of the measured (absolute) slopes of dO_2 (blue triangles) and dCO_2 (red squares) at light with the carbon uptake rate $-(qCO_2 + qHCO_3^-)$ as identified from the experimental data (green diamonds). For the computation of the carbon uptake rate, the quasi steady-state values of qCO_2 and qCO_2 were used (Figure 6 of the main text, right column panels). The full dO_2 and dCO_2 dynamics within Dark-Light-Dark experiment are shown in Figure S6.
